# Quality indicators for structure and process in peri‐operative care: a systematic review

**DOI:** 10.1111/anae.70185

**Published:** 2026-03-11

**Authors:** Sarah Kelly, Paige Cunnington, Harry Dunn, Isla Kuhn, Graham Martin, Mary Dixon‐Woods, Oliver Boney, S. Ramani Moonesinghe, Kristina Wanyonyi‐Kay

**Affiliations:** ^1^ The Healthcare Improvement Studies Institute University of Cambridge Cambridge UK; ^2^ Medical Library University of Cambridge Cambridge UK; ^3^ Department of Anaesthesia and Peri‐operative Medicine University College London Hospitals NHS Foundation Trust London UK; ^4^ Centre for Peri‐operative Medicine, Research Department for Targeted Intervention, Division of Surgery and Interventional Science University College London UK; ^5^ National Institute for Health and Care Research Central London Patient Safety Research Collaboration University College London Hospitals NHS Foundation Trust London UK

**Keywords:** peri‐operative care, quality indicators, quality of health care, safety of health care, systematic review

## Abstract

**Introduction:**

Quality indicators are essential for benchmarking, quality assurance and driving improvement in healthcare. Many indicators exist for peri‐operative care but their relevance and evidence base vary. This systematic review updates a review published 10 years ago. It seeks to identify available structure and process indicators, and assess the level of evidence supporting them, with a view to informing the development of a core indicator set.

**Methods:**

MEDLINE, Embase, CINAHL and the Cochrane Library were searched. English‐language studies in adults were included, alongside grey literature from: clinical, professional and governmental organisations; quality standards; and guidelines.

**Results:**

There were 657 included studies and grey literature sources, alongside indicators from a previously published review. Of a total 615 indicators (324 process indicators, 248 structure indicators and 43 indicators which were not defined clearly but relevant to process or structure), we identified 380 new indicators. Evidence supported 505 (82%) indicators, while 110 (18%) lacked clear evidence. This compared with 47% and 53%, respectively, in the previous review. Only 71 (12%) of the indicators were evaluated for validity. Inconsistencies were noted in definitions, with varying target thresholds reported for the same indicators. Many indicators were developed without the involvement of patients or carers.

**Discussion:**

There is a need for standardisation in the development and naming of peri‐operative quality indicators. Clear reporting, validation and patient involvement would improve their credibility and utility. Rationalising the current large, overlapping number of indicators is essential to enhance usability and ensure meaningful improvement in peri‐operative care.

## Plain Language Summary

**What we did:**

We searched many medical databases and reports to find ways hospitals measure the quality of care around surgery. We included studies and guidelines written in English and looked at reports from health organisations and government groups. We gathered all the different ‘quality indicators’ and checked how much evidence supported each one. We also compared what we found with a similar review done 10 years ago.

**Why did we do it:**

Quality indicators are used to check how well hospitals look after patients and to help improve care. However, there are many different indicators, and not all of them are clear or based on good evidence. We wanted to find out which indicators exist, how strong the evidence is for them and whether they are good enough to help create a smaller, more useful set of indicators for surgery care.

**What we found:**

We found over 600 different quality indicators about care before, during and after surgery. Most of these had some evidence to support them, which is better than 10 years ago. However, only a small number had been properly tested to see if they really work. Many indicators were described in different ways, which makes them hard to compare. We also found that patients and carers were rarely involved in creating these indicators. Overall, there are too many indicators that overlap and cause confusion. We found that quality indicators need clearer definitions, better testing and more patient involvement so they can be more useful for improving care around surgery.

## Introduction

In the UK, the NHS carries out approximately 8 million surgical procedures each year [1] and it is estimated that around 60% of people in England will require surgery at some point in their lives [2]. Globally, it is estimated that at least 321.5 million surgical procedures are needed for a population of about 7 billion [3]. Quality of surgical care is, accordingly, a key imperative and is important in avoiding unwarranted variation and inequities [4, 5, 6]. Critical to quality is sound measurement, typically involving use of indicators that can be classified using the triad of structure, process and outcome, proposed by Avedis Donabedian in the 1960s [7]. Indicators of outcome measure the effects of care on patients, including morbidity and quality of life [8]. Yet they may: evade straightforward measurement; be confounded by case mix and other variables; and not offer actionable information about where improvement efforts should be targeted [7]. Indicators of structure and process therefore have a valuable role in quality measurement, particularly in providing insight into where improvement can be targeted.

Indicators measuring structure are those assessing the settings and systems in which care occurs [8], including features such as: staffing; surgical volume; information system; and facilities. Process indicators measure the activities and actions involved in delivering care, including the activities undertaken in a specific care episode, such as ensuring that patients with diabetes have their blood sugar tested before surgery [9]. All quality indicators need to meet basic criteria and should be: specific; measurable; capable of being recorded routinely; and applicable and transferable across a range of settings [10]. They should be evidence‐based [10] and patients should be involved in their development [11]. However, many quality indicators in healthcare do not meet basic requirements [12, 13, 14] and surgery is no exception.

Peri‐operative care, defined as “*the practice of patient‐centred, multidisciplinary and integrated medical care of patients from the moment of contemplation of surgery until full recovery*” [15], is especially important to outcomes of surgery. Accordingly, sound indicators of structure and process are critical to the ability to monitor quality, identify variations and support improvement in peri‐operative care [16]. A systematic review by Chazapis et al., covering the period up to January 2016, identified multiple challenges (Box 1) [9]. They reported a very large number of indicators of peri‐operative care, but found: they were often duplicative or overlapping; many indicators had a weak evidence‐base; and they lacked the patient perspective.

Box 1 **Findings from the systematic review by Chazapis et al. of structure and process indicators used in peri‐operative care [9].**

- 1282 indicators used in peri‐operative care (819 process and 463 structure) were identified – most from clinical practice guidelines and service evaluations.
- The majority (52.6%) were not evidence‐based. The remainder were built on evidence that ranged from randomised controlled trials to expert opinion.
- Indicators were classified according to the peri‐operative care pathway they focused on: pre‐operative (342, 26.7%); intra‐operative (373, 29.1%); postoperative (227, 17.7%); and spanning the entire pathway (339, 26.4%).
- To address duplication in the initial set, the 1282 indicators were aggregated to produce a final set of 261 indicators (112 structure and 149 process related).
- Process indicators were split relatively evenly across the pre‐, intra‐ and postoperative phases, while approximately half the structure indicators applied across the peri‐operative pathway.
- The 261 indicators focused on different dimensions of the quality and safety of care: effectiveness (136); patient safety (104); efficiency (64); timeliness (30); patient‐centeredness (13); and equity (seven). Some indicators spanned multiple quality domains.

The need for a rationalised, high‐quality set of process and structure indicators for peri‐operative care is now pressing. This is linked to: needs to improve patient safety [17, 18]; efforts to tailor peri‐operative care for high risk groups including older people and those living with frailty [19, 20]; and routine use of enhanced recovery protocols [21]. This systematic review represents the first step of a programme of work (running from August 2023 to June 2025) that aims to develop a core indicator set of process and structure indicators for peri‐operative care. The aims were to: identify which structure and process indicators have been reported for use in peri‐operative care since January 2016 (the period following the review by Chazapis et al. [9]); assess the strength of the evidence supporting each indicator; and to generate an updated list of structure and process indicators and supporting evidence, for both previously identified indicators and those published since 2016.

## Methods

The study was conducted and reported according to PRISMA guidelines [22]. A systematic search was performed in MEDLINE, Embase, CINAHL and the Cochrane Library (1 January 2016 to 8–10 August 2023). The search strategy was limited to studies in English for adult populations (age > 18 y) (online Supporting Information S1 details the full search strategies). Grey literature sources searched included: websites for clinical, professional and governmental organisations involved in peri‐operative care; quality standards and guidelines (including those listed in the Chapazis et al. review [9]); and OpenGrey (online Supporting Information S1).

Articles were eligible for inclusion if they reported the use, development or validation of structure and process indicators of safe and high‐quality peri‐operative care including general anaesthesia or aspects of peri‐operative care relevant to experiences and satisfaction of health service users, carers and healthcare staff. Eligible articles included: empirical studies; review articles; discussion papers; frameworks; organisational case studies; audit projects; surveys; service evaluations; validation studies; clinical, national and professional guidelines; and quality standards (if they reported relevant indicators). Articles were eligible if written in English from any country (including low‐ and middle‐income countries) and in any peri‐operative care setting including (but not limited to): home; outpatient; inpatient; medical wards; and GP settings. Articles were included if they reported indicators or metrics specifically identified as process or structure indicators or indicators of quality judged by the review team to relate to process or structure, even if not explicitly identified as process or structure indicators

For indicators to be included they needed to be relevant to adults (aged > 18 y), with no sex, gender or ethnicity restrictions. They needed to be applicable to all surgical specialities or described for a specific surgical/peri‐operative population or condition but had the potential to be relevant to all surgical specialities. The searches, screening and data extraction took a broad approach, seeking to include any indicators that looked as though they had the potential to be relevant to, and measurable by, all UK hospitals and Trusts providing peri‐operative care and to all specialities, a property we called ‘transferability’. We did not make final decisions on transferability as part of the review, as such decisions will be taken later in the programme of work.

Articles and indicators were not included if they: related solely to care outside the peri‐operative period; were indicators of quality and safety not relevant to the peri‐operative process or structure; or were indicators of outcomes of peri‐operative care. Commentaries, editorials, letters, protocols, clinical case studies and conference abstracts were not included. Numerous recommendations and guidelines were identified (e.g. from clinical and professional organisations) but were not included unless a specific relevant indicator or metric was reported.

To identify studies potentially relevant for inclusion, the titles and abstracts of journal articles were screened independently by two reviewers using Rayyan [23, 24]. Any study marked as ‘include’ by either reviewer was put through to the next stage for full‐text screening and data extraction to check if it reported relevant indicators. Reports identified by the grey literature searches were screened by one reviewer to identify if they contained relevant indicators. Data from included items were extracted into a pre‐defined data extraction form. Indicators and supporting evidence were extracted by one reviewer. A sub‐set of articles from journals and grey literature (10%) were checked by a second reviewer to ensure consistency and agreement of extracted information. The following information was extracted: first author; year of publication; type (journal article or grey literature report); title and DOI and/or reference; process or structure indicators reported with potential relevance to the whole of peri‐operative care; quality indicators that were relevant to process or structure but not specifically identified as process or structure indicators; any description, definition and numerator/denominator or name or title for the indicator; any health condition or surgical subspeciality the indicator was associated with; any information about the stage of peri‐operative care the indicator was associated with (e.g. pre‐operative, postoperative); and any evidence reported to support the quality or validity of the indicator such as supporting evidence from a trial or observational study, a recommendation supporting use of the indicator or a description of how the evidence was obtained. Indicators that were specific to certain health conditions that did not have the potential to be applied across the whole of peri‐operative care were not extracted. Process and structure indicators were, in the first instance, extracted verbatim as reported in the source.

To enable comparability between the analysis by Chazapis et al. [9], we used their approach for assessing the strength of evidence. This was based on the Oxford Centre for Evidence Based Medicine levels of evidence principles [25, 26]. Chazapis et al. did not distinguish between evaluations of the indicators themselves (e.g. to assess validity) and studies that used the indicators as part of a high‐quality study design (e.g. randomised controlled trial).

## Results

**Figure 1:**
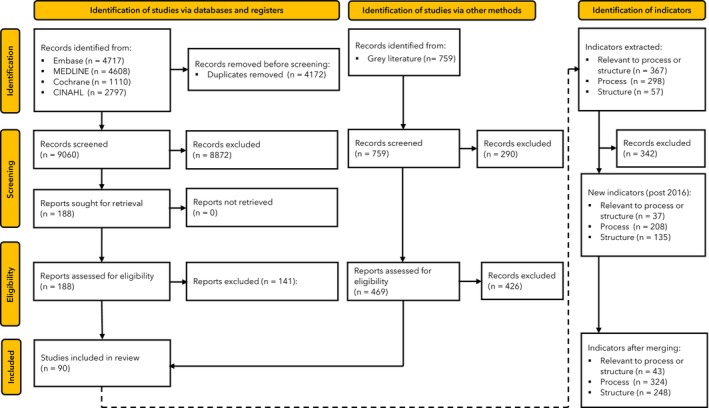
Study flow diagram.

After removal of duplicates, database searches identified 9060 potentially relevant records. Following title and abstract screening, 188 potentially relevant full‐text articles were obtained. Grey literature searches identified 469 potentially relevant reports (Fig. 1). We also identified numerous recommendations and guidelines (e.g. from clinical and professional organisations) but did not include them in the review unless they reported specific relevant indicators or metrics. All full‐texts were screened against our inclusion criteria, resulting in the inclusion of 47 journal articles and 43 grey literature reports. We extracted potentially relevant process or structure indicators meeting the inclusion criteria from the included sources. A number of articles reported multiple indicators. Across the set of articles, we extracted a total of 722 indicators. Of these, 298 were explicitly identified in the literature as process indicators and 57 as structure indicators. We assessed that a further 367 indicators were relevant to process or structure of care but were not explicitly identified in this way in the source articles. Two reviewers classified these 367 indicators as process or structure using definitions of process and structure in the literature [8, 9]. Discrepancies were resolved with advice from peri‐operative clinicians. At this stage, we also reviewed the classification of the 355 indicators explicitly identified as process or structure in the source articles to ensure consistency in categorisation.

We initially extracted indicators exactly as they were worded and reported in the literature. Some indicators were deemed out of scope (i.e. they were not peri‐operative quality indicators), were unclear or lacked sufficient detail or definition. These were removed following an initial face validity check, supported by input from peri‐operative clinicians. Where indicators appeared to be duplicates, we used the most clearly defined and evidenced version.

As the aim of this review was to generate a long list of indicators for a core indicator set by updating a previous review, all potentially transferable indicators were retained at this stage, with the exception of a number of indicators that were considered to be clearly specific to particular health conditions or surgical specialities (and so were evidently not transferable). After this cleaning process, 380 indicators remained.

Indicators were cross‐checked against the final set of indicators in the original review [9]. Once overlapping indicators were removed, the combined set of indicators from the original review and our review comprised a total of 615 indicators (of which 235 were from the previous review). Of these, 324 were, either through identification in the literature or assessment by reviewers, deemed to be process indicators, while 248 were deemed structure indicators and 43 were not defined clearly but were relevant to either process or structure.

Ninety publications were initially included in the review; however, 35 publications were omitted as they reported indicators that were deemed out of scope or were duplicates. Summary characteristics of the remaining 65 included publications [27, 28, 29, 30, 31, 32, 33, 34, 35, 36, 37, 38, 39, 40, 41, 42, 43, 44, 45, 46, 47, 48, 49, 50, 51, 52, 53, 54, 55, 56, 57, 58, 59, 60, 61, 62, 63, 64, 65, 66, 67, 68, 69, 70, 71, 72, 73, 74, 75, 76, 77, 78, 79, 80, 81, 82, 83, 84, 85, 86, 87, 88, 89] are shown in Table 1. The majority were from the UK (27, 42%) and elsewhere in Europe (18, 28%), while 11 (17%) were from the USA and five (8%) were from Australia and New Zealand. Validation studies were the most common, i.e. studies testing indicators against an outcome or evaluating the validity of indicators (14, 22%), followed by audits (10, 15%) and public databases of measures (10, 15%). Other study designs included clinical care standards (nine, 14%), clinical practice guidelines (eight, 12%), reviews (eight, 12%) and service evaluations (six, 9%).

**Table 1:** Included peer‐reviewed publications and grey literature reports, grouped by study design.

Study	Location	Study design	Number of indicators	Clinical area for which study was developed
Antonsen et al. [27]	Denmark	Audit	P: 3, S: 1	N/A
Australian Council on Healthcare Standards [28]	Australia	Audit	P: 2, S: 1	N/A
Australian Council on Healthcare Standards [29]	Australia	Audit	P: 6, U: 2	N/A
Brink et al. [30]	South Africa	Audit	P: 1	N/A
Chereshneva et al. [31]	UK	Audit	P: 45, S: 14, U: 17	N/A
Kristensen et al. [32]	Denmark	Audit	P: 4	Hip fracture
Network for Peri‐operative Critical Care [33]	Ethiopia	Audit	P: 3, S: 2, U: 1	N/A
National Emergency Laparotomy Audit [34]	UK	Audit	P: 6	Emergency laparotomy
PQIP [35]	UK	Audit	P: 10	N/A
Royal College of Physicians [36]	UK	Audit	P: 3	Hip fracture
ANZCA [37]	Australia, New Zealand	Clinical care standard	P: 3, S: 2	N/A
Centre for Peri‐operative Care [38]	UK	Clinical care standard	P: 4, S: 9, U: 1	Invasive procedures
NICE [39]	UK	Clinical care standard	P: 3, S: 6	Joint replacement
NICE [40]	UK	Clinical care standard	P: 4, S: 3	N/A
NICE [41]	UK	Clinical care standard	P: 2, S: 1	Hip fracture
NICE [42]	UK	Clinical care standard	P: 3, S: 12	N/A
Royal College of Surgeons [43]	UK	Clinical care standard	P: 1	Osteoarthritis
Royal College of Surgeons [44]	UK	Clinical care standard	S: 1	Orthopaedic
Royal College of Surgeons [45]	UK	Clinical care standard	P: 1	Orthopaedic
ACSQHC [46]	Australia	Clinical practice guideline	P: 2	N/A
Williams et al. [47]	UK	Clinical practice guideline	S: 2	Gastroenterology
Centre for Peri‐operative Care [48]	UK	Clinical practice guideline	P: 2, S: 2, U: 1	Anaemia
Centre for Peri‐operative Care [49]	UK	Clinical practice guideline	P: 1	OSA
Centre for Peri‐operative Care [50]	UK	Clinical practice guideline	P: 3, S: 1, U: 1	Frailty
Diabetes Technology Network [51]	UK	Clinical practice guideline	P: 1, S: 3	Diabetes
Royal College of Anaesthetists [52]	UK	Clinical practice guideline	P:1, S: 3	Day surgery
Vascular Society [53]	UK	Clinical practice guideline	S: 2	Vascular
American Society of Anesthesiology [54]	USA	Public databases of measures	P: 1, S: 2	Day surgery
American Society of Anesthesiology [55]	USA	Public databases of measures	P: 4	N/A
Anesthesia Quality Institute [56]	USA	Public databases of measures	P: 6, U: 1	N/A
Anesthesia Quality Institute [55]	USA	Public databases of measures	P: 1	N/A
CMS [57]	USA	Public databases of measures	P: 4	N/A
NHS England [58]	UK	Public databases of measures	P: 2	N/A
NHS England [59]	UK	Public databases of measures	P: 1	N/A
NHS England [60]	UK	Public databases of measures	P: 1	N/A
NICE [61]	UK	Public databases of measures	P: 1	Hip fracture
The Joint Commission [62]	USA	Public databases of measures	P: 2	Orthopaedic
Numan et al. [63]	Netherlands	Meta‐analysis	U: 1	Lung cancer
Aggarwal [64]	Canada	Review	U: 1	N/A
Coulson et al. [65]	Australia	Review	S: 1	Cardiac surgery
Erem and Aytac [66]	Turkey	Review	P: 1	N/A
Glarcher et al. [67]	Austria, Germany	Review	P: 10, S: 3, U: 2	Pain
Meissner et al. [68]	Europe, USA	Review	P: 12, S: 4	N/A
Nunes et al. [69]	Portugal	Review	P: 1	Day surgery
Bruno et al. [70]	Madagascar, USA	Service evaluation	S:3, U: 1	N/A
Gimeno‐Moro et al. [71]	Spain	Service evaluation	P: 1	Bariatric surgery
Laurent et al. [72]	France	Service evaluation	P: 1	N/A
NCEPOD [73]	UK	Service evaluation	P: 19, S: 10, U: 1	Diabetes
Bolton NHS Foundation Trust [74]	UK	Service evaluation	P: 3, S: 2	N/A
Panella et al. [75]	Belgium, Italy, Portugal	Service evaluation	P: 2	Hip fracture
Chazapis et al. [9]	UK	Systematic review	P: 116, S: 113, U: 6	N/A
AHRQ [76]	USA	Validation study	P: 8	ERAS
Amini et al. [77]	Netherlands	Validation study	U: 1	Stroke
Barbero et al. [78]	Spain	Validation study	P: 1	ERAS
Chargari et al. [79]	Europe	Validation study	U: 1	Cervical cancer
Chen et al. [80]	China	Validation study	P: 3, S: 15	N/A
Citron et al. [81]	USA	Validation study	P: 5, S: 3, U: 1	N/A
Emond et al. [82]	Netherlands	Validation study	S: 6	N/A
Fischer et al. [83]	Netherlands	Validation study	P: 1, U: 1	Hip replacement
Gencer et al. [84]	Europe	Validation study	U: 1	N/A
Greggi et al. [85]	Italy	Validation study	U: 1	Ovarian cancer
Guzman‐Pruenda et al. [86]	USA	Validation study	P: 1	Colorectal surgery
Leeds et al. [87]	USA	Validation study	P: 1	ERAS
Lian et al. [88]	Norway	Validation study	U: 1	Hip fracture
Tallon Aguilar et al. [89]	Global	Validation study	S: 21	N/A

P, process indicator; S, structure indicator; U, undefined either process or structure; N/A, not applicable.

AJHRQ, Agency for Healthcare Research and Quality; CMS, Centers for Medicare and Medicaid Services; PQIP, Peri‐operative Quality Improvement Programme; ANZCA, Australian and New Zealand College of Anaesthetists; ACSQHC, Australian Commission on Safety and Quality in Healthcare; ERAS, enhanced recovery after surgery; NICE, National Institute for Health and Care Excellence; NCEPOD, National Confidential Enquiry into Patient Outcome and Death; OSA, obstructive sleep apnoea.

Full lists of included indicators are shown in online Supporting Information Tables S1–S3. Indicators were from all phases of the peri‐operative cycle and mostly covered topics related to clinical care protocols, pre‐operative preparation and staff availability. The largest group of indicators (169, 27.5%) concerned the whole peri‐operative pathway, covering aspects of quality such as availability of staff, workforce training and presence of equipment, while the remaining indicators could be categorised into the pre‐ (134, 21.7%), intra‐ (161, 26.2%) and postoperative (151, 24.6%) phases, aligning with the categorisation used by Chazapis et al. [9]. The majority of indicators (549, 89.3%) were not developed for a specific clinical discipline, although some were developed within specific clinical specialties or related to formal standards of care, for example as specified in clinical audits. These indicators were developed for: orthopaedic surgery (31, 5.0%); enhanced recovery after surgery (10, 1.6%); day surgery (eight, 1.3%); emergency laparotomy (six, 1.0%); cardiovascular (four, 0.7%); cancer (three, 0.5%); gastroenterology (two, 0.3%); bariatric (1, 0.2%); and colorectal surgery (one, 0.2%). Patient‐centred indicators (i.e. indicators related to the provision of care that is responsive to patients' preferences, values and needs) [90] were few (8, 1.3%) and focused primarily on shared decision‐making, communication and mechanisms for measuring patient satisfaction. Very few indicators (5, 0.8%) made explicit reference to carers.

The supporting evidence for the included indicators, assessed using the approach by Chazapis et al., was variable. Many (505, 82.1%) were supported by some evidence, defined as either being used in randomised controlled trials, systematic reviews or tested for validity. Of these 505 indicators, 71 (14.1%) had been the subject of studies that evaluated their validity. The remaining 434 (85.9%) indicators had been used as measures in studies ranging in evidence level from level 1a (systematic reviews of randomised controlled trials) to level 5 (expert opinion) (online Supporting Information S1–S3). A sizeable minority (85, 13.8%) of the included indicators were supported by the lowest level of evidence (expert opinion). Many of these indicators concerned activities in the postoperative period. Close to a fifth of indicators (110, 17.9%) did not report information about the level of evidence supporting the indicator in the source article. These indicators were broad in scope and included those applicable to the whole peri‐operative pathway. In comparison, the majority (53%) of indicators found by Chazapis et al. [9] were not supported by evidence. Few indicators appeared to have included input from patients in their development.

## Discussion

Quality of peri‐operative care is important to surgical outcomes [15, 91, 92], but our review suggests that its measurement remains problematic. The large, and likely excessive, number of indicators of structure (n = 112) and process (n = 149) of peri‐operative care identified by Chazapis et al. almost 10 years ago [9] has continued to grow. Our review indicates that there are now 615 indicators (with just 235 of them indicators from the original review by Chazapis et al.), including 324 relating to process and 248 relating to structure. While the substantial increase in the number of published indicators shows growing international interest in monitoring the quality of care [93], the proliferation of measures is unlikely to be warranted and is likely to represent a form of improvement waste [94]. We found that consistent indicator definitions [95] were lacking and some indicators were not categorised as process or structure explicitly, further compounding the entropy we identified. A major concern is that only 71 of the 615 indicators have been evaluated for validity.

Other problems identified by Chazapis et al. have persisted [9]. Reporting of indicator development was often unclear, and information regarding indicator measurability frequently absent. While many of the indicators (82.1%) in our review were supported by some level of evidence, compared with 47.3% in the review by Chazapis et al. [9], more than a fifth of the indicators in our review either lacked information about their evidence base (17.9%) or were developed from expert opinion (14.1%), with limited input from patients or carers. Our review also confirmed that failures of standardisation in the definition and measurement specifications of quality indicators [14] affect peri‐operative indicators, which we found to be reported in slightly different ways across multiple sources or with varying numerical targets (e.g. 85% vs. 90%). Many indicators (n = 367) were not explicitly labelled as process or structure indicators, despite availability of definitions that could help guide classification [8, 9, 96].

Our review underscores the need for a much more strategic approach to quality measurement in peri‐operative care. A co‐ordinated approach to standardisation, consistency of definition, development and reporting of indicators is likely to be of benefit. For peri‐operative care, it will need to tackle the need for indicators to be transferable across diverse surgical disciplines [92, 97].

A strength of this review is its systematic and comprehensive search strategy, which included academic and grey literature. This enhanced the sensitivity of the search and enabled identification of indicators developed and proposed by peri‐operative care professional and regulatory organisations included in clinical standards, guidelines and audit documents. However, the robustness of findings reported in the grey literature is often questionable [98]. The majority of the indicators were derived from high‐income countries and the restriction to English‐language literature may have excluded relevant indicators from other contexts, limiting generalisability [99].

This review has identified a broad range of structure and process indicators but highlights the need for subsequent efforts to develop practical, measurable indicators that align with the complex, multidisciplinary nature of peri‐operative care. Improving measurement of structure and process of peri‐operative care with appropriate indicators can usefully build on methodologies to define valid and patient‐centred peri‐operative care indicators. This approach aligns with programmes like Core Outcome Measures for Peri‐operative and Anaesthetic Care [100]; the Peri‐operative Quality Improvement Programme [101]; and the Standardised Endpoints in Peri‐operative care collaboration [13].

With the now comprehensive long list of indicators, our next steps have been to use participatory approaches, involving healthcare professionals, patients and carers, and consensus‐building to map the indicators to a newly developed conceptual framework of quality and safety in peri‐operative care [102], assess the scope and relevance of the indicators, and agree on a proposed core set of process and structure indicators using a modified COMET methodology.

This review highlights a significant increase in process and structure indicators for peri‐operative care since the last review. However, it also indicates a varied level of evidence supporting these indicators. This underscores the need for improved indicator development methodologies, standardisation and validation to ensure they can be implemented effectively and used to monitor care and drive improvements in peri‐operative care quality and safety.

## Supporting information


**Appendix S1.** Search strategies.

**Table S1.** Structure indicators with level of evidence
**Table S2.** Process indicators with level of evidence.
**Table S3.** Indicators not clearly defined as process or structure with level of evidence.
