# Supplementary material for: Quality indicators for structure and process in peri‐operative care: a systematic review
Source: Anaesthesia. 2026 Mar 11;81(8):1148–59. doi: 10.1111/anae.70185 (PMC13352565; doi:10.1111/anae.70185)
Supplement: Supplementary file 1 — Appendix S1. Search strategies. [file ANAE-81-1148-s002.docx]

**Appendix S1.** Search strategies

**1. Summary of results**

|  | No. of search hits (1^st^ Jan 2016 to run date 8-10^th^ August 2023) |
| --- | --- |
| Medline via Ovid | 4608 |
| Embase via Ovid | 4717 |
| CINAHL via Ebsco | 2797 |
| Cochrane Library | 1110 |
| Total | 13232 |
| **Total deduplicated** | **9060** |

**2. Database search strategies**

# **Medline**

Ovid MEDLINE(R) and Epub Ahead of Print, In-Process, In-Data-Review & Other Non-Indexed Citations, Daily and Versions <1946 to August 08, 2023>

1 (periop* or perop* or peri-op* or per-op* or preop* or pre-op* or postop* or post-op* or intraop* or Intra-op* or (anaesth* or anesth*)).ti,ab. or exp *Perioperative Period/ or exp *Preoperative Care/ or exp *Preoperative Period/ or exp *Intraoperative Care/ or exp *Intraoperative Period/ or exp *Intraoperative Complications/ or exp *Anesthesia, General/ or exp *Postoperative Period/ or exp *Postoperative Care/ 1442672

2 exp *Quality Indicators, Health Care/ or *"Quality of Health Care"/ or exp *Quality Assurance, Health Care/ or *Process Assessment, Health Care/ or *"Standard of Care"/ or (((structure* or process* or quality or safety) adj indicat*) or ((quality or perform* or structure* or process* or safety) adj measur*) or (structure adj (criter* or quality or assess* or healthcare or "health care")) or (quality adj (criter* or assess* or healthcare or "health care" or perform*)) or (process* adj assess*)).ti,ab. or clinical indicator*.ti,ab. 297476

3 1 and 2 10755

4 limit 3 to (english language and yr="2016 -Current") 4608

# **Embase**

Embase <1974 to 2023 August 08>

1 (periop* or perop* or peri-op* or per-op* or preop* or pre-op* or postop* or post-op* or intraop* or Intra-op* or (anaesth* or anesth*)).ti,ab. or *perioperative period/ or *preoperative care/ or *preoperative period/ or *intraoperative period/ or *general anesthesia/ or *postoperative period/ or *postoperative care/ or *Perioperative Care/ 1917369

2 *health care quality/ or *"Standard of Care"/ or (((structure* or process* or quality or safety) adj indicat*) or ((quality or perform* or structure* or process* or safety) adj measur*) or (structure adj (criter* or quality or assess* or healthcare or "health care")) or (quality adj (criter* or assess* or healthcare or "health care" or perform*)) or (process* adj assess*)).ti,ab. or clinical indicator*.ti,ab. 203154

3 1 and 2 8807

4 limit 3 to (english language and yr="2016 -Current") 4729

# **Cochrane Library**

Date Run: 10/08/2023 19:22:50

#1 (((structure* or process* or quality or safety) next indicat*) or ((quality or perform* or structure* or process* or safety) next measur*) or (structure next (criter* or quality or assess* or healthcare or "health care")) or (quality next (criter* or assess* or healthcare or "health care" or perform*)) or (process* next assess*) or clinical indicator*):ti,ab 18887

#2 MeSH descriptor: [Quality Indicators, Health Care] explode all trees 825

#3 MeSH descriptor: [Quality of Health Care] this term only 1156

#4 MeSH descriptor: [Quality Assurance, Health Care] explode all trees 5786

#5 MeSH descriptor: [Process Assessment, Health Care] this term only 332

#6 MeSH descriptor: [Standard of Care] this term only 447

#7 #1 or #2 or #3 or #4 or #5 or #6 26173

#8 (anaesth* or anesth*):ti,ab 97128

#9 (perop* or periop* or peri-op* or per-op* or preop* or pre-op* or postop* or post-op* or intraop* or Intra-op*):ti,ab 175126

#10 MeSH descriptor: [Perioperative Care] this term only 1190

#11 MeSH descriptor: [Perioperative Period] this term only 703

#12 MeSH descriptor: [Preoperative Period] explode all trees 763

#13 MeSH descriptor: [Intraoperative Care] this term only 1762

#14 MeSH descriptor: [Intraoperative Period] this term only 1607

#15 MeSH descriptor: [Intraoperative Complications] this term only 1930

#16 MeSH descriptor: [Postoperative Period] this term only 6230

#17 MeSH descriptor: [Postoperative Care] this term only 5480

#18 MeSH descriptor: [Anesthesia, General] this term only 5602

#19 #9 or #10 or #11 or #12 or #13 or #14 or #15 or #16 or #17 or #18 181309

#20 #7 and #19 with Publication Year from 2016 to 2023, with Cochrane Library publication date Between Jan 2016 and Dec 2023, in Trials 1110

# **CINAHL**

| **#** | **Query** | **Limiters/Expanders** | **Last Run Via** | **Results** |
| --- | --- | --- | --- | --- |
| S1 | (MM "Quality of Health Care") OR (MM "Process Assessment (Health Care)") | Expanders - Apply equivalent subjects  Search modes - Boolean/Phrase | Interface - EBSCOhost Research Databases  Search Screen - Advanced Search  Database - CINAHL | 41,931 |
| S2 | ( TI(((structure* or process* or quality or safety) n1 indicat*) or ((quality or perform* or structure* or process* or safety) n1 measur*) or (structure n1 (criter* or quality or assess* or healthcare or "health care")) or (quality n1 (criter* or assess* or healthcare or "health care" or perform*)) or "process* assess*" or "clinical indicator*") ) OR ( AB(((structure* or process* or quality or safety) n1 indicat*) or ((quality or perform* or structure* or process* or safety) n1 measur*) or (structure n1 (criter* or quality or assess* or healthcare or "health care")) or (quality n1 (criter* or assess* or healthcare or "health care" or perform*)) or "process* assess*" or "clinical indicator*") ) | Expanders - Apply equivalent subjects  Search modes - Boolean/Phrase | Interface - EBSCOhost Research Databases  Search Screen - Advanced Search  Database - CINAHL | 90,624 |
| S3 | S1 OR S2 | Expanders - Apply equivalent subjects  Search modes - Boolean/Phrase | Interface - EBSCOhost Research Databases  Search Screen - Advanced Search  Database - CINAHL | 125,657 |
| S4 | (MM "Postoperative Period") OR (MM "Anesthesia") OR (MM "Postoperative Care") or (MM "Preoperative Care") OR (MM "Preoperative Period") OR (MM "Intraoperative Period") OR (MM "Intraoperative Complications") | Expanders - Apply equivalent subjects  Search modes - Boolean/Phrase | Interface - EBSCOhost Research Databases  Search Screen - Advanced Search  Database - CINAHL | 34,848 |
| S5 | TI(periop* or perop* or peri-op* or per-op* or preop* or pre-op* or postop* or post-op* or intraop* or Intra-op*) or AB (periop* or perop* or peri-op* or per-op* or preop* or pre-op* or postop* or post-op* or intraop* or Intra-op*) | Expanders - Apply equivalent subjects  Search modes - Boolean/Phrase | Interface - EBSCOhost Research Databases  Search Screen - Advanced Search  Database - CINAHL | 226,192 |
| S6 | TI(anaesth* or anesth*) or AB(anaesth* or anesth*) | Expanders - Apply equivalent subjects  Search modes - Boolean/Phrase | Interface - EBSCOhost Research Databases  Search Screen - Advanced Search  Database - CINAHL | 82,285 |
| S7 | S4 OR S5 OR S6 | Expanders - Apply equivalent subjects  Search modes - Boolean/Phrase | Interface - EBSCOhost Research Databases  Search Screen - Advanced Search  Database - CINAHL | 297,037 |
| S8 | S3 AND S7 | Expanders - Apply equivalent subjects  Search modes - Boolean/Phrase | Interface - EBSCOhost Research Databases  Search Screen - Advanced Search  Database - CINAHL | 4,953 |
| S9 | S3 AND S7 | Limiters - Published Date: 20160101-20231231  Expanders - Apply equivalent subjects  Search modes - Boolean/Phrase | Interface - EBSCOhost Research Databases  Search Screen - Advanced Search  Database - CINAHL | 2,859 |
| S10 | S3 AND S7 | Limiters - Published Date: 20160101-20231231  Expanders - Apply equivalent subjects  Narrow by Language: - english  Search modes - Boolean/Phrase | Interface - EBSCOhost Research Databases  Search Screen - Advanced Search  Database - CINAHL | 2,797 |

**3. Grey literature search**

3.1 Google advanced search

3.2 OpenGrey

3.3 Websites/documents

A. UK

B. USA

C. Canada

D. Australia / New Zealand

**A. UK**

- Royal College of Anaesthetists
- Royal College of Surgeons
- National Institute for Health and Care Excellence
- Information Services Division Scotland (now part of Public Health Scotland)
- National Emergency Laparotomy Audit
- National Confidential Enquiry into Patient Outcome and Death
- Healthcare Quality Improvement Partnership datasets
- Centre for Perioperative Care
- National Audit of Breast Cancer in Older Patients
- Perioperative Quality Improvement Programme
- NHS England Commissioning for Quality and Innovation

**B. USA**

- American Society of Anaesthesiologists
- Anaesthesia Quality Institute
- American Medical Association
- Ambulatory Care Quality Alliance
- National Quality Forum
- Centers for Disease Control and Prevention – Surgical Care Improvement Project
- Centers for Medicare and Medicaid Services
- The Joint Commission
- International Quality Indicator Project – Maryland
- Maryland Hospital Association/International Quality Improvement Project
- Veterans Health Administration
- Surgical Care Improvement Project

**C. Canada**

- Canadian Anaesthesiologist Society Guidelines

**D. Australia and New Zealand**

- Australian Council on Healthcare Standards
- National Health and Medical Research Council
- Australian Commission on Safety and Quality in Healthcare Initiative
- Australian New Zealand College of Anaesthetist
