# Supplementary material for: Quality indicators for structure and process in peri‐operative care: a systematic review
Source: Anaesthesia. 2026 Mar 11;81(8):1148–59. doi: 10.1111/anae.70185 (PMC13352565; doi:10.1111/anae.70185)
Supplement: Supplementary file 2 — Table S1. Structure indicators with level of evidence Table S2. Process indicators with level of evidence. Table S3. Indicators not clearly defined as process or structure with level of evidence. [file ANAE-81-1148-s001.docx]

**Table S1.** Structure indicators with level of evidence

| **No.** | **Structure indicators** | **Perioperative phase** | **Level of evidence (studies using indicators as measures)** | **Level of evidence (studies evaluating validity of indicators)** |
| --- | --- | --- | --- | --- |
| 1 | Evidence of local arrangements to ensure that people with iron-deficiency anaemia who are having surgery are offered iron supplementation before surgery **^NEW^** | Pre-op | 2c | - |
| 2 | Evidence of local arrangements to ensure that adults who are having surgery and are expected to have moderate blood loss are offered tranexamic acid **^NEW^** | Pre-op | 1a | - |
| 3 | There is a consultant anaesthetist with responsibility to lead the anaesthetic preoperative assessment service and, this is factored into their job plan | Pre-op | N/A | - |
| 4 | Availability of elderly medicine in the preoperative period for assessments of patients admitted as emergency general surgical patients | Pre-op | 3b | - |
| 5 | There are multidisciplinary team clinics to discuss patients preoperatively | Pre-op | 3b | - |
| 6 | Evidence of local arrangements to ensure that operating teams remove any hand jewellery, artificial nails, and nail polish before starting surgical hand decontamination **^NEW^** | Pre-op | 1a | - |
| 7 | Grading system for determining clinical priority in emergency surgery? **^NEW^** | Pre-op | 2c | - |
| 8 | Hospital policy/guidance on managing operating lists stated that patients with diabetes should be prioritised to be first on the morning or afternoon list? **^NEW^** | Pre-op | 2c | - |
| 9 | Up to date, clear, and complete information about operating lists is immediately available. Any changes are agreed by all relevant parties | Pre-op | 3b | - |
| 10 | Scheduling includes Team Brief time **^NEW^** | Pre-op | 2c | - |
| 11 | There is support for patients with individual or special needs: patients and/or advocates have access to an interpreter | Pre-op | N/A | - |
| 12 | Hospital policy stating that all preoperative investigations should be performed during a single visit **^NEW^** | Pre-op | 2c | - |
| 13 | Access to routine preoperative testing for endemic and epidemic diseases (preoperative testing) **^NEW^** | Pre-op | - | 2a |
| 14 | A pre-op input by a consultant intensivist **^NEW^** | Pre-op | 5 | - |
| 15 | Is water or other clear fluid available for patients to access freely while awaiting surgery? **^NEW^** | Pre-op | 2c | - |
| 16 | Evidence of local arrangements to ensure that people having surgery for which antibiotic prophylaxis is indicated receive this in accordance with the local antibiotic formulary and that this is recorded **^NEW^** | Pre-op | 1a | - |
| 17 | Availability of a protocol on prophylactic antibiotic use **^NEW^** | Pre-op | - | 3a |
| 18 | Evidence of local arrangements to ensure that people having surgery are advised not to remove hair from the surgical site **^NEW^** | Pre-op | 1a | - |
| 19 | Evidence of local arrangements to ensure people having surgery are advised to have (or are helped to have) a shower, bath, or bed bath the day before or on the day of surgery **^NEW^** | Pre-op | 1a | - |
| 20 | General pre-operative assessment clinics available? **^NEW^** | Pre-op | 2c | - |
| 21 | Pre-operative assessment clinic dedicated to the day surgery unit? **^NEW^** | Pre-op | 2c | - |
| 22 | Ability to do preoperative assessment in the community (preoperative assessment) **^NEW^** | Pre-op | - | 2a |
| 23 | Fraction of patients without information of preoperative risk factors **^NEW^** | Pre-op | 5 | - |
| 24 | There is a designated area for private communication with patients. Patients are given adequate information upon which to base their decision about informed consent | Pre-op | 1a | - |
| 25 | There are agreed local policies for preoperative preparation as listed: preoperative fasting, investigations, blood cross-match, thromboprophylaxis, diabetes management, latex allergy, and antacid prophylaxis | Pre-op | 5 | - |
| 26 | Preoperative assessment teams use standard preoperative assessment protocols | Pre-op | 3b | - |
| 27 | Patients admitted for unscheduled surgical care are nursed and managed in a surgical ward or critical care environment | Pre-op | N/A | - |
| 28 | When immediate outcome is dependent on imaging studies (i.e. the patients is to go directly to theatre after imaging) a provisional report is available within 30 min and a definitive report within 1 h | Pre-op | N/A | - |
| 29 | Availability of cardiopulmonary exercise testing for all patients undergoing major surgery | Pre-op | N/A | - |
| 30 | There are agreed specialty risk scoring mechanisms in place and these are applied to all patients admitted as an emergency | Pre-op | N/A | - |
| 31 | Before surgery, except in the case of acute, life-threatening situations, there are clear mechanisms in place, in the absence of patient records, to elicit information from supporters, particularly for unconscious/elderly/confused patients | Pre-op | N/A | - |
| 32 | There is a documented policy to address the airway management of patients in the emergency department | Pre-op | N/A | - |
| 33 | Availability of a protocol on performing prospective risk analysis preoperatively | Pre-op | 5 | - |
| 34 | There are formal protocol/pathways for emergency general surgical patients | Pre-op | 2a | - |
| 35 | Evidence of local arrangements to ensure that adults who will have hip or knee replacement receive advice on preoperative rehabilitation when they are listed for surgery **^NEW^** | Pre-op | 1a | - |
| 36 | Evidence of surgical protocols which include 2 'stop moments' for predefined and systematic checks, during intraoperative formal 'time outs', to confirm that implant details are correct, and all components are compatible **^NEW^** | Intra-op | 1a | - |
| 37 | Evidence of clinical protocols to ensure that adults who are having hip or knee replacement are given tranexamic acid during surgery **^NEW^** | Intra-op | 1a | - |
| 38 | Access to blood and blood conservation techniques (cell salvage or acute normovolaemic haemodilution) are available | Intra-op | N/A | - |
| 39 | Blood storage facilities are in close proximity to emergency theatres and contain O rhesus negative blood | Intra-op | N/A | - |
| 40 | Equipment for fluid and blood warming and rapid infusion is available | Intra-op | N/A | - |
| 41 | Percentage of patients who have consultant (anaesthetists or surgeon) presence in decision making and in theatres **^NEW^** | Intra-op | 2c | - |
| 42 | All patients should have a named and documented supervisory anaesthetist who has overall responsibility for the care of the patient intraoperatively | Intra-op | N/A | - |
| 43 | Is there a responsible person who is nominated to manage and collect data on accidental awareness? **^NEW^** | Intra-op | 2c | - |
| 44 | Clinicians performing endoscopy supported by dedicated endoscopy staff as opposed to other nursing staff (e.g. theatre staff) | Intra-op | N/A | - |
| 45 | Availability of reserved teams to provide planned surgical care (ring-fenced teams) **^NEW^** | Intra-op | - | 2a |
| 46 | Evidence of local arrangements to ensure that operating teams follow practices for surgical hand decontamination **^NEW^** | Intra-op | 1a | - |
| 47 | Evidence of local arrangements to ensure that staff wear specific non-sterile theatre wear in all areas where operations are undertaken **^NEW^** | Intra-op | 1a | - |
| 48 | Evidence of local arrangements to ensure that operating teams minimise any staff movements in and out of the operating area **^NEW^** | Intra-op | 1a | - |
| 49 | Evidence of local arrangements to ensure the existence of surveillance systems that capture inpatient and post-discharge surgical site infections **^NEW^** | Intra-op | 1a | - |
| 50 | Evidence of local arrangements to ensure surveillance data on surgical site infection rates are fed back to relevant staff **^NEW^** | Intra-op | 1a | - |
| 51 | Evidence of local arrangements to ensure that people with a surgical site infection are offered treatment with an antibiotic that covers the likely causative organisms and is selected based on local resistance patterns and the results of microbiological tests **^NEW^** | Intra-op | 1a | - |
| 52 | Sufficient surgical instrument and local sterilisation processes (sterilisation) **^NEW^** | Intra-op | - | 2a |
| 53 | Cross-specialty patient prioritisation for surgery (patient prioritisation) **^NEW^** | Intra-op | - | 2a |
| 54 | Ability to identify and cancel procedures of limited clinical value (procedure prioritisation) **^NEW^** | Intra-op | - | 2a |
| 55 | Fully resourced, dedicated daytime emergency and trauma lists are available | Intra-op | N/A | - |
| 56 | Hospital policy/guidance on managing operating lists? **^NEW^** | Intra-op | 2c | - |
| 57 | Anaesthesia record compliance with ANZCA requirements **^NEW^** | Intra-op | 2a | - |
| 58 | Access to the following facilities with capacity matched to demand, to allow time-critical vascular care to be delivered. Operating theatre, ‘hybrid’ theatre and interventional radiology room – critical care **^NEW^** | Intra-op | 5 | - |
| 59 | In every site where anaesthesia is given, emergency drugs including intralipid, sugammadex, and dantrolene are available and an in-date supply is maintained | Intra-op | N/A | - |
| 60 | Do all anaesthetic machines have an end-tidal volatile alarm enabled as standard? **^NEW^** | Intra-op | 2c | - |
| 61 | Availability of anaesthetic equipment in the operating room: measurement of inspired gas concentrations, saturations, tidal volumes, temperature, non-invasive blood pressure equipment available | Intra-op | N/A | - |
| 62 | There is a planned maintenance and replacement programme for all anaesthetic equipment as required | Intra-op | N/A | - |
| 63 | Access to diagnostics and interventions to identify and treat surgical complications (managing complications) **^NEW^** | Intra-op | - | 2a |
| 64 | Do the logistics of anaesthetic rooms and operating theatres support anaesthesia during patient transfer? **^NEW^** | Intra-op | 2c | - |
| 65 | Percentage of theatres with immediate access to an anaphylaxis treatment pack and management guidelines **^NEW^** | Intra-op | 2c | - |
| 66 | Percentage of theatres containing available anaphylaxis investigation packs **^NEW^** | Intra-op | 2c | - |
| 67 | Formal operational plan to continue planned surgery during external system shocks **^NEW^** | Intra-op | - | 2a |
| 68 | Availability of OR regulations **^NEW^** | Intra-op | - | 3a |
| 69 | Availability of and compliance with local protocol and lists or 'obesity packs' that outline equipment specific for the obese patient and their location in all theatre complexes **^NEW^** | Intra-op | 2c | - |
| 70 | Access to patient-controlled analgesia equipment **^NEW^** | Intra-op | 2c | - |
| 71 | Electronic PCA with parameters, such as bolus dose, can be adjusted as required **^NEW^** | Intra-op | - | 2c |
| 72 | Naloxone is prepared in the department **^NEW^** | Intra-op | - | 2c |
| 73 | Devices for maintaining or raising the temperature of the patient are available including control of theatre temperature | Intra-op | N/A | - |
| 74 | After general or regional anaesthesia, or sedation, all patients recover in a specially designated area which meets AAGBI and Department of Health guidelines (UK) | Intra-op | N/A | - |
| 75 | There are agreed criteria for discharge from recovery | Intra-op | N/A | - |
| 76 | Equipment to provide a full range of local and regional blocks is available in the operating suite | Intra-op | N/A | - |
| 77 | After agreed criteria for discharge have been met, an appropriately trained member of staff accompanies patients during transfer | Intra-op | N/A | - |
| 78 | In a usual week, how many dedicated and planned consultant anaesthetic sessions (i.e. outside of on-call and other duties) support those operating theatres available for adult general surgical emergency cases? | Intra-op | N/A | - |
| 79 | Where sedation is provided by an anaesthetist, there is a policy for the provision of this service in all subspecialty areas and the specifications of the facilities provided | Intra-op | N/A | - |
| 80 | An emergency call system is in place and understood by all relevant staff. Verbal confirmation of the system and how it is used should be given by any member of staff when asked | Intra-op | N/A | - |
| 81 | Drugs intended for regional anaesthesia are stored separately from those intended for i.v. use | Intra-op | N/A | - |
| 82 | There is a written policy for the management of complications of neuraxial blockade | Intra-op | N/A | - |
| 83 | Equipment is available to administer oxygen to all patients undergoing procedures under sedation by an anaesthetist | Intra-op | N/A | - |
| 84 | There is specialized equipment for the management of difficult airways available in every area where anaesthesia is given. The equipment on it should be checked. All members of staff should be able to confirm its location | Intra-op | N/A | - |
| 85 | Facilities for external cardiac pacing are available. Defibrillators should be checked to ensure they include pacing mode | Intra-op | N/A | - |
| 86 | Clinicians wishing to perform ultrasound guided regional anaesthesia should be experienced in the administration of regional nerve blocks and trained in ultrasound guidance techniques | Intra-op | 1b | - |
| 87 | Percentage of staff present in the PACU trained to the recognised standard, audited at different times of day and night **^NEW^** | Post-op | 2c | - |
| 88 | Evidence of local arrangements to ensure that people with iron-deficiency anaemia are offered iron supplementation after surgery **^NEW^** | Post-op | 2c | - |
| 89 | Evidence of the availability of members of the physiotherapy or occupational therapy teams to discuss postoperative rehabilitation with adults who had hip, knee or shoulder replacement and give advice **^NEW^** | Post-op | 1a | - |
| 90 | Availability of postoperative elderly medicine review for postoperative patients | Post-op | 3b | - |
| 91 | Presence of postoperative multidisciplinary consultation for facilitated discharge of patients | Post-op | 3b | - |
| 92 | Percentage of patients where two members of staff (of whom at least one was adequately trained) transferred the patient from recovery **^NEW^** | Post-op | 2c | - |
| 93 | Percentage of conscious patients requiring critical care or critical care monitoring who are being care for in a ratio of one nurse to two patients **^NEW^** | Post-op | 2c | - |
| 94 | Social support system to facilitate safe discharge (social support) **^NEW^** | Post-op | - | 2a |
| 95 | There is provision for appropriate postoperative support including follow-up and outreach after home discharge **^NEW^** | Post-op | 5 | - |
| 96 | Presence of a locally tailored discharge protocol **^NEW^** | Post-op | 2c | - |
| 97 | Delayed discharge due to conversion onto/off CSII **^NEW^** | Post-op | 5 | - |
| 98 | Ability to facilitate timely discharges (timely discharge) **^NEW^** | Post-op | - | 2a |
| 99 | Presence of a surveillance system for postoperative wound infections **^NEW^** | Post-op | - | 3a |
| 100 | Availability of reserved critical care beds for planned surgery (ring-fenced critical care) **^NEW^** | Post-op | - | 2a |
| 101 | Deaths in perioperative patients with diabetes specifically discussed at morbidity and mortality meetings? **^NEW^** | Post-op | 2c | - |
| 102 | There is regular (at least bimonthly) review of all deaths following emergency general surgery | Post-op | N/A | - |
| 103 | Regular education and training of PACU staff to national standards | Post-op | 5 | - |
| 104 | Audit and critical incident systems should be in place in PACU | Post-op | 5 | - |
| 105 | Evidence of local arrangements to ensure that people having surgery and their carers receive information and advice on wound and dressing care, including how to recognise problems with the wound and who to contact if they are concerned **^NEW^** | Post-op | 1a | - |
| 106 | Evidence of local arrangements to ensure that adults who have had hip, knee or shoulder replacement receive advice on postoperative rehabilitation before they are discharged from hospital **^NEW^** | Post-op | 1a | - |
| 107 | Evidence of local arrangements to ensure that people with hip fracture start rehabilitation at least once a day, no later than the day after surgery **^NEW^** | Post-op | 1a | - |
| 108 | Are there protocols in the hospital for escalation of opioids and non-opioid rescue for poor pain control? **^NEW^** | Post-op | 2c | - |
| 109 | Availability of written pain management standards **^NEW^** | Post-op | 2c | - |
| 110 | SOPs/protocols (management of acute pain in the post-operative setting) **^NEW^** | Post-op | 2c | - |
| 111 | Policies of pain assessment are required, and 4 elements of pain assessment/reassessment are emphasised, including intensity, location, quality and time characteristics of occurrence **^NEW^** | Post-op | - | 2c |
| 112 | Policies of pain treatment are required, including 1) the goal of pain treatment; 2) its principles; 3) nonpharmacological treatment; 4) analgesia treatment; and 5) side effects of treatment **^NEW^** | Post-op | - | 2c |
| 113 | Policies of patient pain education are required, including 1) the usage of pain assessment tools; 2) the importance of postoperative pain management; 3) the usage of nonpharmacological treatment; 4) effects of pharmacological intervention and its side effects; 5) misconceptions about pain management; 6) continuous education to patients during the perioperation **^NEW^** | Post-op | - | 2c |
| 114 | Availability of pain management services **^NEW^** | Post-op | 2c | - |
| 115 | Creation of a 24/7 acute pain service **^NEW^** | Post-op | 2c | - |
| 116 | There is specialized equipment for the management of postoperative pain. An adequate number of PCAs epidural pumps and the arrangements for their use should be available for the services being provided | Post-op | N/A | - |
| 117 | There is a need for patient information boards that present knowledge of pain management **^NEW^** | Post-op | - | 2c |
| 118 | A handbook is prepared for patient education, which can be used by the patient to learn about pain management **^NEW^** | Post-op | - | 2c |
| 119 | Documentation of pain management nursing including 1) pain intensity; 2) pain location; 3) quality of pain; 4) pain assessment tools; 5) time characteristics of pain; and 6) influence of pain on daily life **^NEW^** | Post-op | - | 2c |
| 120 | PACU bed area, capacity, and equipment are all maintained to national standards | Post-op | 5 | - |
| 121 | Transfer from operating room to PACU is with a formal handover process | Post-op | 5 | - |
| 122 | There is a policy for the post-procedural review of all patients: surgical and anaesthetic | Post-op | 3b | - |
| 123 | Patients and supporters are given clear information on discharge from the service and are able to make contact with a healthcare professional for advice and support | Post-op | N/A | - |
| 124 | Availability of inpatient and post-discharge rehabilitation | Post-op | N/A | - |
| 125 | Availability of surgical follow up within 30 days following hospital discharge | Post-op | 1a | - |
| 126 | Each PACU unit should have suitable recovery and discharge criteria | Post-op | 5 | - |
| 127 | Surgical bed occupancy rate **^NEW^** | All | 5 | - |
| 128 | Number of specialist surgical, anaesthetic, and obstetric physicians who are working, per 100,000 population **^NEW^** | All | 2a | - |
| 129 | Number of Full Time Equivalent (FTE) specialist qualified Surgeon, Anesthesiologist and Obstetrician (SAO) number at a facility/catchment population ∗100,000 **^NEW^** | All | - | 2a |
| 130 | Number of accredited surgeon professionals | All | 2a | - |
| 131 | Number of accredited anaesthesia professionals | All | 2a | - |
| 132 | Perioperative team size and composition | All | 2a | - |
| 133 | Proportion of the population that can access, within 2 h, a facility that can do caesarean section, laparotomy, and treatment of open fracture (the Bellwether procedures) **^NEW^** | All | 2a | - |
| 134 | Travel time to hospital – proportion of patients interviewed reporting having travelled <2h to reach hospital **^NEW^** | All | - | 2a |
| 135 | Anaesthetists confirm that staffing numbers, with regard to anaesthesia assistants, are commensurate with needs as recommended by applicable guidelines and staff have the competence to perform requisite tasks **^NEW^** | All | 5 | - |
| 136 | Team-based training and education for each standard **^NEW^** | All | 2c | - |
| 137 | Process of training and quality management **^NEW^** | All | 2c | - |
| 138 | Formal staff training in: use of equipment, clinical practice guidelines, technical and non-technical skills of perioperative care | All | 3b | - |
| 139 | There is a trained resuscitation team for adults | All | N/A | - |
| 140 | Adequate surgeon training and experience for each specialty in compliance with national training | All | 2b | - |
| 141 | Availability of hospital guideline for detection and management of anaemia applicable to the perioperative setting **^NEW^** | All | 1a | - |
| 142 | Protocol for the recognition and management of hypo and hyperglycaemia **^NEW^** | All | 2c | - |
| 143 | Hospital protocol for VRII? **^NEW^** | All | 2c | - |
| 144 | Protocols exist for the perioperative management of: venous thromboembolism prophylaxis, avoidance of hypothermia, management of diabetes mellitus, handover, anaesthetic emergencies, morbidly obese patients, handling of complains, elderly patients, remote site anaesthesia, end of life care, and critical care referral | All | 1b | - |
| 145 | All perioperative services are consultant led | All | N/A | - |
| 146 | Pharmacists are readily available to consult with nurses and medics on non-critical care units; pharmacy formularies are accessible | All | 3b | - |
| 147 | The emergency surgical service has an identified medical and nurse lead (separate to the leads of elective surgery) | All | N/A | - |
| 148 | Dedicated surgical scrub nurses for each surgical specialty are present | All | N/A | - |
| 149 | Suitable administrative and secretarial support is available at all times for the emergency surgical team | All | N/A | - |
| 150 | Availability of a team to support/deliver perioperative management of anaemia **^NEW^** | All | 1a | - |
| 151 | Availability of a perioperative frailty team such as Perioperative Medicine for Older People undergoing surgery (POPS) team **^NEW^** | All | 5 | - |
| 152 | Named lead for perioperative diabetes? **^NEW^** | All | 2c | - |
| 153 | Availability of specialist services: burn care, transplant, trauma, ERCP, prosthetics, brachytherapy, radiotherapy, sexual function, specialist continence, psychological counselling, diabetes nurse specialist, physiotherapy, and acute medical admissions | All | 3b | - |
| 154 | A senior manager is directly responsible for day surgery **^NEW^** | All | 5 | - |
| 155 | Ability to redistribute staff within and between hospitals to maintain capacity (staff redistribution) **^NEW^** | All | - | 2a |
| 156 | Nursing ratios **^NEW^** | All | 2c | - |
| 157 | Availability of a perioperative antibiotic protocol | All | 5 | - |
| 158 | Emergency (2 h) surgical access **^NEW^** | All | 5 | - |
| 159 | Presence of agreed protocols to defer elective activity in order to give adequate priority to unscheduled admissions | All | N/A | - |
| 160 | Availability of protocols and equipment to appropriately manage the brain in older adults **^NEW^** | All | 2c | - |
| 161 | Protocols exist for the perioperative management of: venous thromboembolism prophylaxis, avoidance of hypothermia, management of diabetes mellitus, handover, anaesthetic emergencies, morbidly obese patients, handling of complains, elderly patients, remote site anaesthesia, end of life care, and critical care referral | All | 1b | - |
| 162 | Availability of elderly medicines on site. Routine daily assessment of surgical patients? | All | N/A | - |
| 163 | Presence or absence of language line/translation **^NEW^** | All | 5 | - |
| 164 | Printed patient information and alternative language leaflets available | All | N/A | - |
| 165 | Availability of reserved planned surgery theatres (Ring-fenced theatres) **^NEW^** | All | - | 2a |
| 166 | Availability of reserved surgery beds (ring-fenced beds) **^NEW^** | All | - | 2a |
| 167 | How many operating theatres in the hospital? (excluding radiology suites, dedicated obstetric, minor ops but including day case theatres) | All | 3b | - |
| 168 | Number of general surgical beds: the number of funded level 2 and 3 beds available for adult (>18) general surgical patients | All | N/A | - |
| 169 | Bed size of hospital: how many adult inpatient/overnight/23 h stay available within the hospital | All | 1b | - |
| 170 | Reliable supply of electricity (electricity supply) **^NEW^** | All | - | 2a |
| 171 | Reliable supply of supplementary oxygen (oxygen supply) **^NEW^** | All | - | 2a |
| 172 | Reliable supply and management of essential perioperative drugs (drug supply) **^NEW^** | All | - | 2a |
| 173 | Reliable supply and management of devices and implants (device supply) **^NEW^** | All | - | 2a |
| 174 | Availability of protective measures for theatre teams (protective equipment) **^NEW^** | All | - | 2a |
| 175 | Ability to transfer patients to another hospital with greater capacity (hospital transfer) **^NEW^** | All | - | 2a |
| 176 | Flexibility to rearrange hospital areas to provide a segregated pathway for planned surgery (flexible areas) **^NEW^** | All | 2a | - |
| 177 | New equipment risk assessment and ratification processes in place **^NEW^** | All | 2c | - |
| 178 | Availability of a protocol on (performing) prospective risk analysis of medical equipment **^NEW^** | All | - | 3a |
| 179 | 24 h availability of diagnostic and interventional radiology | All | N/A | - |
| 180 | Capacity to use telephone or video calls for outpatient appointments (remote outpatient appointments) **^NEW^** | All | - | 2a |
| 181 | 24-hour telephone availability of a member of the arthroplasty team **^NEW^** | All | 1a | - |
| 182 | Day surgery patients should have access to a 24 h staffed telephone line for advice and help | All | 4 | - |
| 183 | Day surgery exists as a separate and ‘ring-fenced’ administrative care pathway **^NEW^** | All | 5 | - |
| 184 | Having sufficient capacity to deliver ERCP 52 weeks a year **^NEW^** | All | 5 | - |
| 185 | Regular morbidity and mortality meetings that are able to demonstrate outcomes that meet minimum standards **^NEW^** | All | 5 | - |
| 186 | Morbidity and mortality meeting. Morbidity and mortality meeting conducted, documented as conducted. Minimum of 9 per year **^NEW^** | All | - | 3a |
| 187 | Presence of a morbidity and mortality registration **^NEW^** | All | - | 3a |
| 188 | Standards are explicitly addressed in trust-wide and local (e.g., site/specialty) policies and procedures **^NEW^** | All | 2c | - |
| 189 | Governance reviews for all standards **^NEW^** | All | 2c | - |
| 190 | Processes in place for qualitative assessment and review of engagement with all standards **^NEW^** | All | 2c | - |
| 191 | Count boards standardised across areas with standardised documentation and symbols **^NEW^** | All | 2c | - |
| 192 | The purpose of standards are to maximise patient safety, improve patient satisfaction, support best clinical practice, reduce cost to the Trust relating to litigation and complaints, and contribute to reduced length of stay **^NEW^** | All | 5 | - |
| 193 | Every trust should have a local management plan in place based on these or other authoritative guidelines **^NEW^** | All | 5 | - |
| 194 | Anaesthesia care is provided by registered medical practitioners who have completed the ANCA training program or ANZCA-associated program or who have been assessed by ANZCA **^NEW^** | All | 5 | - |
| 195 | Medical specialists trained in comprehensive geriatric assessment (CGA) are available to review patients both pre- and post-operatively (self-declaration) **^NEW^** | All | 5 | - |
| 196 | Formal staff training in: use of equipment, clinical practice guidelines, technical and non-technical skills of perioperative care | All | 3b | - |
| 197 | Adequate surgeon training and experience for each speciality in compliance with national training guidance | All | 2b | - |
| 198 | All hospital trusts should have clear, audited perioperative pathways from pre-assessment through to discharge. These should broadly be in line with NCEPOD recommendations **^NEW^** | All | 5 | - |
| 199 | The service submits data to prescribed national audits. Regular audit of critical incidents | All | N/A | - |
| 200 | Clinical audit of all emergency surgical procedures whether undertaken in an operating theatre or another area (e.g. emergency resus room) is regularly undertaken | All | N/A | - |
| 201 | Presence of IV placement protocol **^NEW^** | All | 5 | - |
| 202 | Debrief log and action log is kept **^NEW^** | All | 2c | - |
| 203 | Availability of a protocol on responsibilities regarding maintenance of medical equipment **^NEW^** | All | - | 3a |
| 204 | Proportion of households protected against impoverishment from direct out-of-pocket payments for surgical and anaesthesia care **^NEW^** | All | 2a | - |
| 205 | Evidence of local arrangements to ensure that people having surgery under general anaesthesia have normothermia maintained before, during (unless active cooling is part of procedure) and after surgery **^NEW^** | All | 1a | - |
| 206 | Availability of a perioperative antibiotic protocol | All | 5 | - |
| 207 | There is a guideline for the prevention and management of delirium in the perioperative period **^NEW^** | All | 1a | - |
| 208 | Availability of a perioperative anticoagulant protocol | All | 5 | - |
| 209 | Regular revision of guidelines **^NEW^** | All | 2c | - |
| 210 | Specific regulations regarding the multidisciplinary pain service team are recorded in the hospital document, and they include 1) service scope; 2) service time (24 hours on duty, serving at night and on weekends); 3) formulating and updating guidelines and regulations for analgesic treatment; 4) carrying out training for doctors; 5) carrying out training for nurses; 6) a nurse with a clinical specialization in pain included in the multidisciplinary pain service team; and 7) carrying out quality improvement projects regularly **^NEW^** | All | - | 2c |
| 211 | Policies of staff training are required, including 1) organizing continuing education courses regularly; 2) organizing a multidisciplinary training course; 3) orientation; 4) post-training examination **^NEW^** | All | - | 2c |
| 212 | The department has a funded and staffed acute pain service | All | 2b | - |
| 213 | Dedicated pain nurses **^NEW^** | All | 2c | - |
| 214 | A multidisciplinary pain service team should be established, and it should include specialized pain nurses and doctors **^NEW^** | All | - | 2c |
| 215 | The ratio of the number of on-the-job nurses in departments to that of open beds is calculated **^NEW^** | All | - | 2c |
| 216 | Nurses who are experienced and knowledgeable in pain management are essential to ensure good quality of pain management **^NEW^** | All | - | 2c |
| 217 | Regular training programs **^NEW^** | All | - | 2c |
| 218 | Anaesthetists and other healthcare providers should use standardized, validated instruments to facilitate the regular evaluation and documentation of pain intensity, the effects of pain therapy, and side effects caused by the therapy | All | 2b | - |
| 219 | 24 h availability of: X-ray, computed tomography, ultrasound, isotope bone scan, multiparametric magnetic resonance imaging, teleradiology, reporting by radiologist, reporting by specialised radiologist | All | 3b | - |
| 220 | Availability of: malignant hyperthermia kit, difficult intubation kit, and cardiac arrest cart in the theatre complex | All | N/A | - |
| 221 | 24 h availability of: biochemistry, haematology, microbiology, and blood bank laboratories | All | N/A | - |
| 222 | Surgical on-call rota is in compliance with national guidance | All | N/A | - |
| 223 | Availability of appropriate facilities for rest and refreshment. Availability of consultant within 30 min of base site | All | 2b | - |
| 224 | Rotas should be provided and include the allocation of formal handover time and place as well as which staff should be present at this handover | All | N/A | - |
| 225 | Does the hospital accept emergency surgical admissions? | All | N/A | - |
| 226 | 24h availability of diagnostic and interventional radiology | All | N/A | - |
| 227 | The service has mechanisms to receive feedback from patients and supporters. Printed patient information and alternative language leaflets available | All | N/A | - |
| 228 | Availability of dedicated office space, swipe card access, admin staff, and skilled assistance for surgical staff | All | N/A | - |
| 229 | University affiliation of the general surgery subsection | All | 3b | - |
| 230 | Theatre suite conforms to Department of Health building standards | All | N/A | - |
| 231 | Presence of a formal handover process for consultants and non-consultant clinicians | All | N/A | - |
| 232 | Accreditation of the surgical unit by the joint commission or cancer commission | All | 3b | - |
| 233 | Dedicated operating rooms are available for each surgical speciality | All | 5 | - |
| 234 | There is adequate protection provided for staff in hazardous situations | All | N/A | - |
| 235 | Are admitted patients retained by the on-call consultant or are they handed over? Is there a formal handover policy? | All | N/A | - |
| 236 | All research is R and D reviewed and REC reviewed. Opportunities to engage in research are prioritized by the unit/network | All | N/A | - |
| 237 | There is a resuscitation officer responsible for coordinating and training of staff | All | N/A | - |
| 238 | Anaesthetists offering perioperative analgesia services should provide, in collaboration with other healthcare professionals as appropriate, on-going education in analgesia | All | 2b | - |
| 239 | The presence of centralization of hospital specialties | All | 2b | - |
| 240 | Presence of appropriate operating room equipment in compliance with national standards | All | 3b | - |
| 241 | Does the hospital participate in clinical trials | All | 3b | - |
| 242 | Does the hospital disseminate reports to its community on quality and costs of healthcare services | All | 3b | - |
| 243 | Modified early warning scores are used on surgical wards | All | N/A | - |
| 244 | There is a defined governance structure to assure the quality of the service and allow for continuous improvement | All | N/A | - |
| 245 | Senior clinicians are involved in the discussion of end of life pathways. Written policy should be provided as well as a verbal account of discussions of end of life pathways | All | N/A | - |
| 246 | A representative range of resuscitation equipment, matching that in use and including mannequins, is available for training purposes by the resuscitation training officer | All | N/A | - |
| 247 | There is a local resuscitation policy in compliance with national guidelines | All | N/A | - |
| 248 | Surgical specialty under which amputation was performed (vascular, general, foot, and ankle surgeons) | All | N/A | - |

***Note.*** ANZCA = Australian and New Zealand College of Anaesthetists; AAGBI = Association of Anaesthetists of Great Britain and Ireland; CSII = continuous subcutaneous insulin infusion; ERCP = endoscopic retrograde cholangiopancreatography; NCEPOD = National Confidential Enquiry into Patient Outcome and Death; OR = operating room; PCA = patient-controlled anaesthesia; PACU = post-anaesthesia care unit; SOP = standard operating procedure; VRII = variable rate intravenous insulin. **^NEW^** = indicators reported for use since January 2016.

**Table S2**. Process indicators with level of evidence

| **No.** | **Process indicators** | **Perioperative phase** | **Level of evidence (studies using indicators as measures)** | **Level of evidence (studies evaluating validity of indicators)** |
| --- | --- | --- | --- | --- |
| 1 | Proportion of people with iron-deficiency anaemia who are having surgery and receive iron supplementation before surgery **^NEW^** | Pre-op | 1a | - |
| 2 | Percentage of patients, aged 18 years and older, undergoing elective total joint arthroplasty who were screened for anaemia preoperatively AND, if positive, have documentation that one or more of the following management strategies were used prior to PACU discharge **^NEW^** | Pre-op | 1a | - |
| 3 | Anaemia screening and treatment for all patients undergoing major elective surgery **^NEW^** | Pre-op | 1a | - |
| 4 | Proportion of patients preoperatively screened, treated, and followed up for anaemia **^NEW^** | Pre-op | 1a | - |
| 5 | Preoperative glucose monitoring for patients with diabetes mellitus is undertaken | Pre-op | 2b | - |
| 6 | Percentage of elective patients with diabetes who are managed by simple manipulation of existing medication if the anticipated starvation time is only one missed meal **^NEW^** | Pre-op | 2c | - |
| 7 | Diabetes medications documented on the day of surgery **^NEW^** | Pre-op | 2c | - |
| 8 | Written instructions for specific medicines are handed out to patients preoperatively. This includes information on: anticoagulants, diabetic medications, cardiovascular medications, and hormonal medications | Pre-op | 5 | - |
| 9 | Presence of an up to date medication list is documented in the medical record | Pre-op | 3b | - |
| 10 | Percentage of primary care referrals containing all recommended information (this includes HbA1c less than 69mmol.mol-1, blood pressure, BMI, estimated glomerular filtration rate and details of patients’ diabetes management **^NEW^** | Pre-op | 2c | - |
| 11 | Information of the management of the patient’s diabetes in the community was available at the GP referral? **^NEW^** | Pre-op | 2c | - |
| 12 | A recent (3 months prior to surgery) HbA1c was available at the pre-operative assessment clinic? **^NEW^** | Pre-op | 2c | - |
| 13 | Percentage of patients with diabetes referred from surgical outpatients to preoperative assessment **^NEW^** | Pre-op | 2c | - |
| 14 | Percentage of patients with diabetes who are listed in the first third of the operating list (morning or afternoon) **^NEW^** | Pre-op | 2c | - |
| 15 | Percentage of patients for whom a perioperative diabetes plan is created at the preoperative assessment clinic **^NEW^** | Pre-op | 2c | - |
| 16 | Documented evidence that the patient was given specific instructions on the management of their diabetes prior to surgery? **^NEW^** | Pre-op | 2c | - |
| 17 | Documented evidence that the patient was included in their diabetes management plan? **^NEW^** | Pre-op | 2c | - |
| 18 | Documented plan for the management of their diabetes recorded on the day of surgery? **^NEW^** | Pre-op | 2c | - |
| 19 | Preoperative assessment is undertaken by staff familiar with the day surgery pathway **^NEW^** | Pre-op | 2c | - |
| 20 | Pre-anaesthesia optimisation involving a collaborative and timely multidisciplinary approach is advisable **^NEW^** | Pre-op | 4 | - |
| 21 | Chlorhexidine cloth and wash the night before and the day of operation **^NEW^** | Pre-op | - | 2b |
| 22 | Proportion of patients who have a chlorhexidine shower preoperatively | Pre-op | 2b | - |
| 23 | People having surgery are advised not to remove hair from the surgical site and to have a shower/bath the day on or before surgery | Pre-op | N/A | - |
| 24 | Elapsed time between admission and entry into operating theatre is measured | Pre-op | 3b | - |
| 25 | Day of surgery admission **^NEW^** | Pre-op | N/A | - |
| 26 | Hip fracture patients are admitted under the joint care of a consultant geriatrician and a consultant orthopaedic surgeon | Pre-op | N/A | - |
| 27 | System for confirming that relevant investigations and resuscitation had been completed and the patient was fit for surgery **^NEW^** | Pre-op | 2c | - |
| 28 | Percentage of patients with documentation of a healthcare proxy, living will, or advanced care directives prior to surgery **^NEW^** | Pre-op | - | 3a |
| 29 | Proportion of patients considered high risk assessed and investigated preoperatively **^NEW^** | Pre-op | 2c | - |
| 30 | The proportion of all patients having elective or emergency surgery who have their perioperative risk explicitly recorded in the consent form and medical notes **^NEW^** | Pre-op | 2c | - |
| 31 | Proportion of surgeons and anaesthetists including risk of perioperative neurocognitive disorders in the informed consent process **^NEW^** | Pre-op | 2c | - |
| 32 | Proportion of patient bookings accompanied by a risk prediction **^NEW^** | Pre-op | 2c | - |
| 33 | Percentage of patients who underwent a non-emergency surgery who had their personalized risks of postoperative complications assessed by their surgical team prior to surgery using a clinical data-based, patient-specific risk calculator and who received personal discussion of those risks with the surgeon **^NEW^** | Pre-op | 1b | - |
| 34 | Proportion of patients in whom a risk assessment was documented preoperatively **^NEW^** | Pre-op | 2c | - |
| 35 | Routine use of risk scoring systems prior to surgery? **^NEW^** | Pre-op | 2c | - |
| 36 | A pre-operative assessment of risk was made on admission? **^NEW^** | Pre-op | 2c | - |
| 37 | Proportion of patients with a documented risk assessment prior to theatre **^NEW^** | Pre-op | 2c | - |
| 38 | Documented risk assessment before surgery **^NEW^** | Pre-op | 2c | - |
| 39 | Proportion of obese patients (a) screened for OSA; (b) assessed by a clinician for OSA; (c) managed according to risk stratification **^NEW^** | Pre-op | 2c | - |
| 40 | Each patient should have his or her expected risk of death estimated and documented prior to intervention and due adjustments made in urgency of care and seniority of staff involved | Pre-op | 1b | - |
| 41 | What proportion of patients undergoing elective major surgery have an objective measurement of fitness **^NEW^** | Pre-op | 2c | - |
| 42 | Patients who completed the general health and joint specific functional status assessments within 90 days (and/or within 365 days) prior to surgery **^NEW^** | Pre-op | N/A | - |
| 43 | Pre-anaesthesia consultation completed by anaesthetist? **^NEW^** | Pre-op | 5 | - |
| 44 | Patient assessed by an anaesthetist on the day of surgery? **^NEW^** | Pre-op | 2c | - |
| 45 | Percentage of patients assessed preoperatively for risk of PONV **^NEW^** | Pre-op | 2c | - |
| 46 | Percentage of patients regardless of age undergoing a total knee or total hip replacement who are evaluated for the presence or absence of venous thromboembolic and cardiovascular risk factors within 30 days prior to the procedure **^NEW^** | Pre-op | 2c | - |
| 47 | Proportion of pre-operative assessment and/or operating lists that includes the patients' weight and BMI **^NEW^** | Pre-op | 2c | - |
| 48 | Early screening – identify patients in need of optimisation early, and at the latest when they are added to a surgical waiting list **^NEW^** | Pre-op | 1a | - |
| 49 | Preoperative methicillin-resistant Staphylococcus aureus patient screening is undertaken and documented | Pre-op | N/A | - |
| 50 | Each patient should have appropriate preoperative tests: haemoglobin or haematocrit, platelets, sodium, potassium, chloride, glucose, urea, creatinine, chest C-ray, height, and weight | Pre-op | 3b | - |
| 51 | Preoperative assessment clinic - telephone contact with patient within 7 days of admission **^NEW^** | Pre-op | 1a | - |
| 52 | What resources are available to patients attending preoperative assessment clinics to encourage lifestyle changes and how they can be accessed? **^NEW^** | Pre-op | 2c | - |
| 53 | Are all smokers referred to a local smoking cessation service (aim for more than 80%)? **^NEW^** | Pre-op | 2c | - |
| 54 | If there is an exercise intervention programme available, what proportion of high-risk patients are offered access to this intervention? **^NEW^** | Pre-op | 2c | - |
| 55 | What written fasting instructions are given to patients in the preoperative phase? **^NEW^** | Pre-op | 2c | - |
| 56 | Is there information on the walls that patients and their carers may see to reinforce the guidance around fasting? **^NEW^** | Pre-op | 2c | - |
| 57 | Proportion of patients who have received physiotherapy assessment **^NEW^** | Pre-op | 2c | - |
| 58 | The proportion of patients on admitted pathways for: cardiac surgery; cardiothoracic surgery; neurosurgery; AAA surgery; and within cardiology; TAVI and complex cardiac devices; who receive a priority categorisation **^NEW^** | Pre-op | 5 | - |
| 59 | Number of patients who received a patient information leaflet about pain relief **^NEW^** | Pre-op | 2c | - |
| 60 | Patients and/or their advocates are given information about the possible side effects of pain relief drugs | Pre-op | 2a | - |
| 61 | Written preoperative information about postoperative pain **^NEW^** | Pre-op | 4 | - |
| 62 | Identify patients at risk of severe pain **^NEW^** | Pre-op | 5 | - |
| 63 | Cases with signed informed consent paperwork or documentation that informed consent was obtained or unable to be obtained because of patient condition **^NEW^** | Pre-op | - | 1a |
| 64 | Informed consent documentation **^NEW^** | Pre-op | - | 2c |
| 65 | Percentage of patients receiving PONV prophylaxis as per local guidelines **^NEW^** | Pre-op | 2c | - |
| 66 | Pre-emptive administration of droperidol or dexamethasone is beneficial for preventing or ameliorating PONV **^NEW^** | Pre-op | 2c | - |
| 67 | Percentage of patients with a documented discharge plan prior to their surgery. This should involve medical, nursing, physiotherapist and occupational health staff **^NEW^** | Pre-op | 2c | - |
| 68 | Proportion of surgical procedures for which antibiotic prophylaxis is indicated for which the person having surgery receives antibiotic prophylaxis in accordance with the local antibiotic formulary and that this is recorded **^NEW^** | Pre-op | 1a | - |
| 69 | Proportion of surgical site infections for which the person with the infection receives treatment with an antibiotic that covers the likely causative organisms and is selected based on local resistance patterns and the results of microbiological tests **^NEW^** | Pre-op | 1a | - |
| 70 | Consultant surgeon review before surgery | Pre-op | 3b | - |
| 71 | What proportion of patients was reviewed by a consultant surgeon within 12h of emergency presentation at hospital | Pre-op | 4 | - |
| 72 | Percentage of patients who have received an anaesthetic assessment before the day of surgery | Pre-op | 1b | - |
| 73 | The following medical history should be documented in the medical record prior to the operation: past medical history, past surgical history, drug history, allergies | Pre-op | 2a | - |
| 74 | Adequate preoperative fasting: clear fluids up to 2 h prior to surgery, solids up to 6 h prior to surgery | Pre-op | 1b | - |
| 75 | Time from diagnosis/referral to operation should be <2 months | Pre-op | 3b | - |
| 76 | All patients, on admission, receive an assessment of venous thromboembolism and bleeding risk using risk assessment criteria | Pre-op | 5 | - |
| 77 | Patient nutritional status assessed within 48 h of admission to hospital by a dietician | Pre-op | 1b | - |
| 78 | Mechanical bowel prep not used routinely for colonic surgery | Pre-op | 1a | - |
| 79 | Performance of risk assessment for pressure ulcers using a standardized scale upon admission | Pre-op | 3b | - |
| 80 | Cancer care plan intent documented in the medical notes: curative, palliative, or no active treatment (supportive) | Pre-op | N/A | - |
| 81 | Elderly patients should have a pre op mobility and cognitive assessment | Pre-op | N/A | - |
| 82 | If a patient is to undergo intestinal surgery, then the plan for surgery should be communicated to the referring physician and the patient's primary care physician | Pre-op | 2a | - |
| 83 | Days from fracture injury to admission to hospital measured and documented | Pre-op | 3b | - |
| 84 | Percentage of patients with malignancy who undergo adjuvant chemotherapy preoperatively | Pre-op | 5 | - |
| 85 | Percentage of patients/carers who are offered verbal and written information on venous thromboembolism prevention as part of their hospital admission process | Pre-op | N/A | - |
| 86 | Patients provided with anti-embolism stockings have them fitted and monitored in accordance with National Institute for Health and Care Excellence guidance | Pre-op | N/A | - |
| 87 | Elapsed time between admission/referral to when first seen by consultant surgeon is measured and documented | Pre-op | N/A | - |
| 88 | The maternity team is notified when a pregnant woman is admitted with a non-obstetric surgical problem | Pre-op | N/A | - |
| 89 | If the patient was admitted with ischaemia or diabetic foot sepsis, did a consultant vascular surgeon review them within 24 h of admission? | Pre-op | N/A | - |
| 90 | Was the patient seen by an amputation/discharge coordinator preoperatively | Pre-op | N/A | - |
| 91 | Patient seen by inpatient acute pain team preoperatively | Pre-op | N/A | - |
| 92 | Stoma care - patients with colorectal cancer who require a stoma are assessed and have their stoma site marked preoperatively by a nurse with expertise in stoma care | Pre-op | N/A | - |
| 93 | Any changes to surgical lists are agreed by all relevant parties | Pre-op | N/A | - |
| 94 | Proportion of hip, knee, or shoulder replacement operations during which there are 2 ‘stop moments’ to check implant details and the compatibility of all components **^NEW^** | Intra-op | 1a | - |
| 95 | Timeout performed for any surgery or procedure **^NEW^** | Intra-op | 5 | - |
| 96 | 100% of records have documented SBYB check and intravenous cannula insertion **^NEW^** | Intra-op | 2c | - |
| 97 | Percentage of anaesthetists & anaesthetic assistants who report always performing SBYB **^NEW^** | Intra-op | 2c | - |
| 98 | Proportion of surgical procedures on adults under general or regional anaesthesia in which the person having surgery has their core temperature measured and documented in accordance with NICE guideline **^NEW^** | Intra-op | 1a | - |
| 99 | Adults having surgery under general or regional anaesthesia have normothermia (temperature >36 degrees) maintained before, during, and after surgery | Intra-op | 1a | - |
| 100 | Achieving 40% (or fewer) patients still receiving IV antibiotics past the point at which they meet switching criteria **^NEW^** | Intra-op | 1a | - |
| 101 | Proportion of adults who are having surgery and are expected to have moderate blood loss who receive tranexamic acid **^NEW^** | Intra-op | 1a | - |
| 102 | Percentage of eligible patient cases where tranexamic acid was used **^NEW^** | Intra-op | 1a | - |
| 103 | Proportion of patients who had moderate blood loss given tranexamic acid intraoperatively **^NEW^** | Intra-op | 1a | - |
| 104 | Where surgery has an expected blood loss of >500 ml, tranexamic acid should be administered **^NEW^** | Intra-op | 1a | - |
| 105 | Proportion of hip or knee replacement operations during which tranexamic acid is given **^NEW^** | Intra-op | 1a | - |
| 106 | Percentage of eligible cases where cell salvage was used **^NEW^** | Intra-op | 2c | - |
| 107 | Proportion of patients who are bleeding tested appropriately intra- and postoperatively **^NEW^** | Intra-op | 2c | - |
| 108 | Intraoperative blood loss is measured and recorded | Intra-op | 2b | - |
| 109 | In hip replacement cases, the number of operations in which patients did not receive transfusion of homologue blood (as one component of ERAS process measures) **^NEW^** | Intra-op | - | 5 |
| 110 | Avoided blood product administration during surgery (as one component of ERAS process measures) **^NEW^** | Intra-op | - | 5 |
| 111 | Blood availability ratio **^NEW^** | Intra-op | 5 | - |
| 112 | Fraction of patients with non-emergency bleeding where haemoglobin level is measured prior to erythrocyte transfusion **^NEW^** | Intra-op | 5 | - |
| 113 | Capillary blood glucose was measured in the theatre recovery area? **^NEW^** | Intra-op | 2c | - |
| 114 | Percentage of patients who receive hourly monitoring of blood glucose during their procedure and in recovery **^NEW^** | Intra-op | 2c | - |
| 115 | Percentage of patients aged 18 years and older who undergo elective inpatient surgery and who have a blood glucose level of >180mg/dL after anaesthesia start time and who receive insulin during anaesthesia or PACU care **^NEW^** | Intra-op | 2a | - |
| 116 | Percentage of patients aged 18 years and older, with a diagnosis of diabetes mellitus receiving anaesthesia services in an ambulatory setting who experienced a blood glucose level >180 mg/dL who received insulin prior to anaesthesia end time AND had a follow-up glucose check prior to discharge **^NEW^** | Intra-op | 2a | - |
| 117 | VRIII commenced on admission? **^NEW^** | Intra-op | 2c | - |
| 118 | Percentage of people in whom a variable rate intravenous insulin infusion is appropriately used **^NEW^** | Intra-op | 2c | - |
| 119 | Maintenance of euglycaemia perioperatively: use of standardized protocol to maintain serum glucose <200 mg day-1 | Intra-op | 2b | - |
| 120 | Adequate perioperative management of patient's current medications | Intra-op | 1b | - |
| 121 | Percentage of patients, regardless of age, that undergo an open interventional pain procedure for whom ALL of the following infection control best practices are followed by anaesthesiologist(s) and scrub technologist(s), in addition to standard sterile technique: (a) double gloving, (b) chlorhexidine with alcohol used for surgical site preparation, (c) weight-based pre-operative antibiotic dosing and, if indicated by procedure duration, weight-based re-dosing, (d) administration of pre-operative antibiotics within 1 hour, or 2 hours for vancomycin, prior to surgical incision **^NEW^** | Intra-op | 1a | - |
| 122 | Surgical field preparation with chlorhexidine-alcohol | Intra-op | 1b | - |
| 123 | Prophylactic antibiotics are administered within 60 min before start of surgery | Intra-op | 1a | - |
| 124 | If hair removal is required, it should not be performed with a razor but with clippers | Intra-op | 1a | - |
| 125 | Proportion of patients who have had appropriate prophylactic antibiotic selection for surgical patients | Intra-op | 1a | - |
| 126 | Proportion of patients arriving in theatre within a time recorded as appropriate for the urgency of surgery (this metric assesses the interval between decision to operate, and arrival in theatre) **^NEW^** | Intra-op | 2c | - |
| 127 | The proportion of people with hip fracture who receive surgery on the day of, or the day after, admission **^NEW^** | Intra-op | 1a | - |
| 128 | Is the date of surgery the same day of the day following first presentation with hip fracture **^NEW^** | Intra-op | 1a | - |
| 129 | Surgery by the day following presentation with hip fracture **^NEW^** | Intra-op | 1a | - |
| 130 | Proportion of admitted patients with a hip fracture who received surgery within 36 hours of presentation to first hospital **^NEW^** | Intra-op | 1a | - |
| 131 | Proportion of patients arriving in theatre within a time appropriate for the urgency of surgery: immediate surgery for bleeding, surgery underway <3 hours for septic shock, <6 hours in sepsis source control or <18 hours in other cases **^NEW^** | Intra-op | 2c | - |
| 132 | Arrival to theatre in a timescale appropriate for the urgency of surgery **^NEW^** | Intra-op | 2c | - |
| 133 | Time from emergency department presentation to non-elective abdominal surgery **^NEW^** | Intra-op | - | 2b |
| 134 | Hours from ED arrival to non-elective abdominal surgery **^NEW^** | Intra-op | - | 2b |
| 135 | The time interval between induction and surgeon’s arrival **^NEW^** | Intra-op | 5 | - |
| 136 | Achieving priority categorisation of patients within selected surgery and treatment pathways according to clinical guidelines **^NEW^** | Intra-op | 2c | - |
| 137 | Median [interquartile range (IQR)] time (hours) between hospital arrival and antibiotic administration amongst those with sepsis on admission **^NEW^** | Intra-op | 2c | - |
| 138 | Delays/Cancellations/workarounds due to problems in implant processes **^NEW^** | Intra-op | 2c | - |
| 139 | Percentage of patients who were administered patient-controlled analgesia **^NEW^** | Intra-op | - | 3a |
| 140 | Percentage of patients who were administered local wound analgesia **^NEW^** | Intra-op | - | 3a |
| 141 | Intravenous analgesia (patient controlled analgesia or i.v. lidocaine) | Intra-op | 4 | - |
| 142 | Percentage of patients requiring anaesthesia services with a documented assessment of neuromuscular blockade reversal after last dose of non-depolarizing neuromuscular blockade **^NEW^** | Intra-op | 2c | - |
| 143 | Percentage of patients aged 12 years and older, requiring anaesthesia services where non-depolarizing neuromuscular blockade is used and neostigmine, sugammadex, and/or edrophonium are administered prior to extubation **^NEW^** | Intra-op | 2c | - |
| 144 | Proportion of patients with a hip fracture who received a nerve block prior to surgery **^NEW^** | Intra-op | 1a | - |
| 145 | Postanaesthetic transfer of care: use of a checklist or protocol for direct transfer of care from procedure room to intensive care | Intra-op | N/A | - |
| 146 | Percentage of patients who had a minimally invasive surgical approach **^NEW^** | Intra-op | - | 3a |
| 147 | Surgical procedures with a predicted mortality >10% should be conducted under direct supervision of consultant surgeon and anaesthetist | Intra-op | 3b | - |
| 148 | Number of recognised episodes of actual or near miss incorrect site procedures **^NEW^** | Intra-op | 2c | - |
| 149 | Number of actual/near miss wrong implant events **^NEW^** | Intra-op | 2c | - |
| 150 | Number of recognised failed reconciliation events **^NEW^** | Intra-op | 2c | - |
| 151 | Are all cases of awareness reported as critical incidents and reviewed? **^NEW^** | Intra-op | 2c | - |
| 152 | Percentage of patients having lactate measurement and goal directed fluid therapy in theatres **^NEW^** | Intra-op | 2c | - |
| 153 | Optimized perioperative fluid management: targeting cardiac output, avoiding over-hydration, and judicious use of vasopressors. Targeted fluid therapy using the Doppler is recommended | Intra-op | 1b | - |
| 154 | Measurement and documentation of pain intensity scores after major surgery | Intra-op | 1b | - |
| 155 | Timely access to any analgesia **^NEW^** | Intra-op | 3a | - |
| 156 | Timely access to parenteral opioid analgesia **^NEW^** | Intra-op | 3a | - |
| 157 | Patients receiving appropriate analgesic dose **^NEW^** | Intra-op | 3a | - |
| 158 | Patients receiving analgesic by appropriate route **^NEW^** | Intra-op | 3a | - |
| 159 | % of multimodal analgesia **^NEW^** | Intra-op | - | 1a |
| 160 | Optimal analgesia (VAS ≤ 4) (ERAS pathway for bariatric surgery) **^NEW^** | Intra-op | - | 2b |
| 161 | Percentage of patients aged 18 years and older, who undergo a procedure under an inhalational general anaesthetic, AND who have three or more risk factors for PONV, who receive combination therapy consisting of at least two prophylactic pharmacologic antiemetic agents of different classes preoperatively and/or intraoperatively **^NEW^** | Intra-op | 5 | - |
| 162 | A multimodal approach for postoperative nausea and vomiting prophylaxis should be adopted in all patients with ≥2 risk factors | Intra-op | 1b | - |
| 163 | Proportion of surgical cases performed in the operating room during which the full WHO Safe Surgery Checklist, or expanded equivalent, is performed **^NEW^** | Intra-op | - | 2b |
| 164 | Rate of compliance with administration within 60 min **^NEW^** | Intra-op | 5 | - |
| 165 | Wound catheters or transversus abdominis planeblock used for intraoperative analgesia | Intra-op | 2b | - |
| 166 | Type of anaesthesia administered documented | Intra-op | N/A | - |
| 167 | An appropriately trained and experienced anaesthetist is present throughout the conduct of all general and regional anaesthesia for operative procedures | Intra-op | 1a | - |
| 168 | Number of cancelled planned operations | Intra-op | 4 | - |
| 169 | Duration of surgery measured and documented | Intra-op | 2b | - |
| 170 | Recommended standards of monitoring are met for each patient. This should be visible on the anaesthetic chart | Intra-op | N/A | - |
| 171 | Appropriate surgical approach for current operative procedure used | Intra-op | 2b | - |
| 172 | Patients for whom a central venous catheter was inserted with all elements of sterile barrier technique followed Documentation of daily examination of line site for signs of infection and continued need for central line | Intra-op | 1a | - |
| 173 | Mechanical thromboprophylaxis used intraoperatively | Intra-op | 5 | - |
| 174 | Percentage of 1st cases starting on time measured and recorded | Intra-op | 3b | - |
| 175 | Operating room turnover time (min) measured | Intra-op | 3b | - |
| 176 | Critically ill patients in the recovery area are cared for by appropriately trained staff and have appropriate monitoring and support | Intra-op | N/A | - |
| 177 | Measures to ensure proper positioning on table documented to prevent peripheral nerve damage and maintain skin integrity | Intra-op | 3b | - |
| 178 | Induction time (min) and emergence time (min) are recorded | Intra-op | 3b | - |
| 179 | Intraoperative use of forced air warming | Intra-op | 4 | - |
| 180 | Surgical pathology specimens are correctly labelled: labelled, filled container, correct laterality, correct tissue type, patient name, correct patient name | Intra-op | 2b | - |
| 181 | Multimodal approach to optimizing postoperative gut function is used | Intra-op | 2b | - |
| 182 | Surgeons use explicit procedure specific intraoperative checklists | Intra-op | 3b | - |
| 183 | Perioperative urine output monitored carefully in patients with renal failure | Intra-op | 5 | - |
| 184 | All anaesthetic equipment is checked before use according to AAGBI published guidelines and the checks are documented | Intra-op | N/A | - |
| 185 | Physician Quality Reporting System/Surgical Care Improvement Project documentation available and completed | Intra-op | N/A | - |
| 186 | People with hip fracture have their schedule on a planned trauma list, with consultant or senior staff supervision | Intra-op | N/A | - |
| 187 | Proportion of procedures complying to the relevant ERAS protocol (where available) **^NEW^** | Post-op | - | 2c |
| 188 | Percentage of patients who tolerated weight bearing on the first postoperative day or sooner **^NEW^** | Post-op | - | 3a |
| 189 | Proportion of patients being mobilised at least once a day **^NEW^** | Post-op | 2c | - |
| 190 | Performance of active mobilization within 24-48 hours in patients who can walk before fracture **^NEW^** | Post-op | 1b | - |
| 191 | DrEaMing in the first 24h after surgery **^NEW^** | Post-op | 2b | - |
| 192 | Perioperative carbohydrate loading for abdominal/colorectal/gynaecological/urological and thoracic surgery **^NEW^** | Post-op | 2c | - |
| 193 | Reducing use of nasogastric tubes and abdominal drains **^NEW^** | Post-op | 2b | - |
| 194 | Percentage of patients having a full medicines reconciliation within 24 hours of admission to the EPC facility **^NEW^** | Post-op | 5 | - |
| 195 | Proportion of people with iron-deficiency anaemia who receive iron supplementation after surgery **^NEW^** | Post-op | 1a | - |
| 196 | Percentage of patients aged 18 years and older, with a current diagnosis of diabetes mellitus receiving anaesthesia services for office-based or ambulatory surgery who experienced a blood glucose level ≥180 mg/dL who received education on managing their glucose in the postoperative period prior to discharge **^NEW^** | Post-op | 2a | - |
| 197 | Postoperative treatment of diabetes mellitus (or documentation of attempt) to keep BM <10 mmol litre-1 on day of surgery and the first 2 postoperative days | Post-op | 1b | - |
| 198 | Patients discharged to home following total hip or total knee replacement **^NEW^** | Post-op | 5 | - |
| 199 | Place of discharge from hospital **^NEW^** | Post-op | 5 | - |
| 200 | Is the patient known to have been discharged to their original home or care home or be there at 120-day follow-up? **^NEW^** | Post-op | 5 | - |
| 201 | Percentage of patients assessed for discharge readiness using the protocol **^NEW^** | Post-op | 2c | - |
| 202 | Percentage of patients not meeting discharge criteria who received anaesthetic review prior to discharge **^NEW^** | Post-op | 2c | - |
| 203 | Time taken for the anaesthetists to review patients not meeting discharge criteria after being contacted and reasons for delay **^NEW^** | Post-op | 2c | - |
| 204 | Percentage of patients discharged from recovery to a general ward who are satisfied discharge criteria **^NEW^** | Post-op | 2c | - |
| 205 | Percentage of patients not meeting discharge criteria who were discharged to a safe clinical area **^NEW^** | Post-op | 2c | - |
| 206 | Assessment of functional level at discharge **^NEW^** | Post-op | 5 | - |
| 207 | Post discharge rehabilitation program **^NEW^** | Post-op | 5 | - |
| 208 | Percentage of patients, regardless of age, who received anaesthesia services in an ambulatory setting whose post-discharge status was assessed within 72 hours of discharge **^NEW^** | Post-op | 5 | - |
| 209 | Patients are discharged with medications prescribed for use as part of their post procedural management as well as their normal medications that they may normally be taking, along with a discharge plan that includes specific instructions **^NEW^** | Post-op | 5 | - |
| 210 | Discharge letter with information about home medication (older geriatric hip fracture patients) **^NEW^** | Post-op | 1b | - |
| 211 | Provision of medication prescriptions to the patient at discharge (surgical quality improvement projects and their relations with VTE) **^NEW^** | Post-op | 5 | - |
| 212 | Proportion of hip fracture operations after which the person starts rehabilitation no later than the day after surgery **^NEW^** | Post-op | 1a | - |
| 213 | Proportion of hip fracture operations after which the person has rehabilitation at least once a day **^NEW^** | Post-op | 1a | - |
| 214 | Proportion of adults who had hip, knee or shoulder replacement who receive advice on postoperative rehabilitation during a discussion with a member of the physiotherapy or occupational therapy team, before discharge from hospital **^NEW^** | Post-op | 1a | - |
| 215 | Post discharge rehabilitation program **^NEW^** | Post-op | 5 | - |
| 216 | Length of hospital stay of patients undergoing major surgery who have needed an intervention (e.g., blood transfusion) to treat their anaemia **^NEW^** | Post-op | 1a | - |
| 217 | Length of hospital stay in patients CFS≥5 **^NEW^** | Post-op | 2c | - |
| 218 | Percentage of patients with length of stay > 21 days with CFS≥5 **^NEW^** | Post-op | 2c | - |
| 219 | Length of hospital stay of patients undergoing major surgery who have anaemia **^NEW^** | Post-op | 1a | - |
| 220 | PACU length of stay measured | Post-op | 3b | - |
| 221 | Review by a specialist from Elderly Medicine in the postoperative period | Post-op | 1a | - |
| 222 | Percentage of opioid-naïve (not taking outpatient opioids within 10 days prior to surgery) patients prescribed an opioid analgesic in the discharge orders/instructions **^NEW^** | Post-op | - | 3a |
| 223 | Percentage of patients who had two or more non-opioid analgesics in the postoperative period within 48 hours of surgery end **^NEW^** | Post-op | - | 3a |
| 224 | Proportion of patients with a postoperative risk of death ≥5% who were directly admitted to critical care postoperatively **^NEW^** | Post-op | 2c | - |
| 225 | The portion of patients considered high-risk (predicted hospital mortality greater than 5%) undergoing surgery not admitted to intensive care **^NEW^** | Post-op | 2c | - |
| 226 | Structured assessment of patient mortality and morbidity risk, carried out at the end of surgery | Post-op | N/A | - |
| 227 | Percentage of patients aged 18 years or older, who undergo an elective procedure requiring anaesthesia services who are screened for obstructive sleep apnoea, AND, if positive, for whom two or more selected mitigation strategies were used prior to PACU discharge **^NEW^** | Post-op | 2c | - |
| 228 | Act early planning postoperative destination **^NEW^** | Post-op | 2c | - |
| 229 | Percentage of patients who are visited postoperatively by an anaesthetist **^NEW^** | Post-op | 2c | - |
| 230 | Anaesthetists contribute to advanced postoperative care **^NEW^** | Post-op | 5 | - |
| 231 | Percentage of unconscious patients who are being cared for on a one-to-one basis **^NEW^** | Post-op | 2c | - |
| 232 | Percentage of patients having their observations recorded with appropriate frequency **^NEW^** | Post-op | 2c | - |
| 233 | Percentage of patients with complete documentation of observations from PACU arrival until discharge **^NEW^** | Post-op | 2c | - |
| 234 | Documented patient handover – operating suite to recovery area **^NEW^** | Post-op | 5 | - |
| 235 | Documented patient handover – recovery area to ward **^NEW^** | Post-op | 5 | - |
| 236 | Relief of respiratory distress in the recovery period **^NEW^** | Post-op | 5 | - |
| 237 | Unplanned stay in recovery room >2 hours **^NEW^** | Post-op | 5 | - |
| 238 | Unplanned ICU admission within 24 hours after procedure **^NEW^** | Post-op | 5 | - |
| 239 | Pain intensity scores recorded regularly for surgical patients **^NEW^** | Post-op | 1b | - |
| 240 | Patients with monitoring of pain using a scale during their stay (documented scores) **^NEW^** | Post-op | 4 | - |
| 241 | Patients with monitoring of pain using a scale (frequency during stay) **^NEW^** | Post-op | 4 | - |
| 242 | Pain documented in medical record **^NEW^** | Post-op | 4 | - |
| 243 | Pain assessment **^NEW^** | Post-op | 3a | - |
| 244 | Patients with any documented pain assessment **^NEW^** | Post-op | 3a | - |
| 245 | Patients with documented pain assessment (validated pain score) **^NEW^** | Post-op | 3a | - |
| 246 | Patients with physician-documented pain assessment **^NEW^** | Post-op | 3a | - |
| 247 | Patients with documented pain reassessment after treatment **^NEW^** | Post-op | 3a | - |
| 248 | Timeliness of pain assessment **^NEW^** | Post-op | 3a | - |
| 249 | Timely reassessment of pain relief after treatment **^NEW^** | Post-op | 3a | - |
| 250 | Pain assessment documented before discharge **^NEW^** | Post-op | 3a | - |
| 251 | Postoperative pain assessed using visual analogue scale at (a) PACU and (b) 48 hours post-op **^NEW^** | Post-op | 5 | - |
| 252 | Postoperative pain evaluation and follow-up (at rest or movement, after analgesic) **^NEW^** | Post-op | 4 | - |
| 253 | Patients with pain rated 0/10 at discharge **^NEW^** | Post-op | 3a | - |
| 254 | Routine postoperative pain assessment and assessment intervals **^NEW^** | Post-op | 4 | - |
| 255 | Postoperative pain assessments should be performed with each set of vital signs | Post-op | 5 | - |
| 256 | Percentage of patients, aged 18 years and older, undergoing selected surgical procedures that were managed with multimodal pain medicine **^NEW^** | Post-op | 1a | - |
| 257 | Severe pain not responding to pain protocol in the recovery period **^NEW^** | Post-op | 5 | - |
| 258 | % of postoperative visit (postoperative visit refers to departments not having sufficient staff (such as anesthesiologists, members of multidisciplinary organizations, etc.) to perform pain-related follow-up on postoperative patients) **^NEW^** | Post-op | - | 2c |
| 259 | Application of consensus guidelines and protocols for post-surgical pain treatment **^NEW^** | Post-op | 4 | - |
| 260 | Number of patients monitored for postoperative pain and visited by an anaesthetist or by a trained medical person **^NEW^** | Post-op | 4 | - |
| 261 | Fraction of patients with severe postoperative pain (numeric rating scale >7) in the postoperative observation unit **^NEW^** | Post-op | 5 | - |
| 262 | Percentage of patients receiving treatment of PONV as per local guidelines **^NEW^** | Post-op | 2c | - |
| 263 | Fraction of patients with nausea requiring treatment in the postoperative observation unit **^NEW^** | Post-op | 5 | - |
| 264 | Daily anaesthetist review following epidural analgesia | Post-op | N/A | - |
| 265 | Proportion of surgical patients who had an order for venous thromboembolism prophylaxis to be given within 24 h before incision/after surgery end | Post-op | 1a | - |
| 266 | Proportion of patients whose prophylactic antibiotics were discontinued within 24 h after surgery end time | Post-op | 1a | - |
| 267 | Discharge needs assessment, venous thromboembolism prophylaxis, rehab, and follow up are organized postoperatively for patients | Post-op | 1a | - |
| 268 | Urinary catheter removed on Postoperative Day 1 or Postoperative Day 2 with day of surgery being Day 0, or reason for continuing use documented | Post-op | 1b | - |
| 269 | Postoperative delirium screening for all patients | Post-op | 1a | - |
| 270 | People having surgery and their carers receive information and advice on wound and dressing care | Post-op | 2a | - |
| 271 | Cognitive and functional assessment performed daily postoperatively and at discharge | Post-op | 3b | - |
| 272 | Official PACU to ward handover undertaken for all patients | Post-op | 5 | - |
| 273 | Patient’s condition and vital signs evaluated continuously in the PACU | Post-op | 5 | - |
| 274 | Stimulation of bowel movements using an even fluid balance, laxatives and chewing gum | Post-op | 1b | - |
| 275 | Percentage of recovery nurses following acute pain protocols | Post-op | 5 | - |
| 276 | Early warning system used on postoperative wards | Post-op | 2b | - |
| 277 | Immediately post-surgery a member of the medical/nursing team updates the patient’s supporter(s) of the outcome of surgery | Post-op | N/A | - |
| 278 | Postanaesthesia documentation is documented to the agreed national standard | Post-op | N/A | - |
| 279 | Visual phlebitis scores are measured daily postoperatively | Post-op | 2b | - |
| 280 | The head of the bed is elevated postoperatively | Post-op | 2b | - |
| 281 | Hydration, pressure care, assessment and treatment of pain, and attention to nutrition and continence are begun in the emergency room and are continued in the orthopaedic ward postoperatively | Post-op | 3b | - |
| 282 | Patients having a postoperative physician review (not critical care) | Post-op | 3b | - |
| 283 | Waiting time from time appointed for surgical procedure until discharge | Post-op | 3b | - |
| 284 | Days from surgery until discharge from hospital | Post-op | 3b | - |
| 285 | Patients receiving prescribed antiemetic treatment when nausea and vomiting are present during acute pain management | Post-op | 4 | - |
| 286 | Time from operation until adjuvant chemotherapy | Post-op | 5 | - |
| 287 | All patients given supplemental oxygen as required | Post-op | 5 | - |
| 288 | At the end of surgery, was the decision made to place the patient on an end of life pathway; was this documented | Post-op | N/A | - |
| 289 | What proportion of patients were admitted directly to a high dependency unit or intensive therapy unit following surgery | Post-op | N/A | - |
| 290 | After fracture surgery there is communication with the physicians responsible for post-surgical care | Post-op | N/A | - |
| 291 | Shared Decision Making: Proportion of patients engaging in shared decision making, measured as far as practical, possibly qualitatively **^NEW^** | All | 5 | - |
| 292 | Percentage of patients who received tranexamic acid perioperatively **^NEW^** | All | - | 1a |
| 293 | Percentage of patients aged 18 years and older, with a current diagnosis of diabetes mellitus receiving anaesthesia services for office-based or ambulatory surgery who received insulin perioperatively and who received a follow-up blood glucose level check following the administration of insulin and prior to discharge **^NEW^** | All | 2a | - |
| 294 | Percentage of diabetic patients aged 18 years and older, who receive an office-based or ambulatory surgery whose blood glucose level is appropriately managed throughout the perioperative period **^NEW^** | All | 2a | - |
| 295 | Multidisciplinary team involved in all hip fracture patients > 70 years **^NEW^** | All | 2b | - |
| 296 | Cooperation between orthopaedic, physicians, and anaesthetists in preoperative, operative, and postoperative medical management, and in the rehabilitation of hip fracture patients | All | 2b | - |
| 297 | Daily operating room usage **^NEW^** | All | - | 2b |
| 298 | Delay for elective surgical admission **^NEW^** | All | N/A | - |
| 299 | Percentage of patients aged 70 years or older, who undergo an inpatient procedure requiring anaesthesia services and have a positive frailty screening result who receive a multidisciplinary consult or care during the hospital encounter **^NEW^** | All | 1a | - |
| 300 | Proportion of patients aged ≥65 years and frail or ≥80 years who were assessed by a member of the geriatrician-led multidisciplinary team during any part of the perioperative pathway **^NEW^** | All | 1a | - |
| 301 | Use of Bauer survey or pomVLAD for in-patient stay **^NEW^** | All | N/A | - |
| 302 | Longer term outcomes – use the EQ-5D-5L and WHO Disability Assessment Schedule (quality of life questionnaires) **^NEW^** | All | N/A | - |
| 303 | Percentage of at-risk patients receiving care modified to reduce their cognitive risk **^NEW^** | All | 2c | - |
| 304 | What proportion of patients have had health-related quality of life questionnaire? **^NEW^** | All | 2c | - |
| 305 | Assessment of frailty using a validated scoring system in all patients aged over 65 **^NEW^** | All | 2c | - |
| 306 | Percentage of patients aged 75 years and over screened for delirium before admission and on each postoperative day **^NEW^** | All | 2c | - |
| 307 | Are patients with difficult airway given adequate information and the Difficult Airway Society airway alert card? **^NEW^** | All | 2c | - |
| 308 | Patient satisfaction with inpatient management of CSII therapy ‘loss’ of insulin pumps removed from patients **^NEW^** | All | 2c | - |
| 309 | Responsiveness to inpatients personal needs – measured by Overall experience whilst in hospital **^NEW^** | All | 5 | - |
| 310 | The percentage of patients with an advanced airway in place who have continuous capnography monitoring **^NEW^** | All | 2c | - |
| 311 | Perioperative pain recorded (at rest or movement) **^NEW^** | All | 4 | - |
| 312 | SCQIPP Questionnaire (communication, action, trust, environment) **^NEW^** | All | 4 | - |
| 313 | Chronic beta blocker use is continued in perioperative period (24 h before incision to first 2 postoperative days) | All | 1a | - |
| 314 | Surgery takes place during standard daytime working hours (including weekends) except in exceptional circumstances | All | 3b | - |
| 315 | Documentation of oral intake during the hospitalisation | All | 3b | - |
| 316 | Perioperative continued use of aspirin for patients with drug-eluting coronary stents | All | N/A | - |
| 317 | Information is provided to patients and supporters at each stage of the care pathway. Communication with patients and supporters is consultant-led | All | N/A | - |
| 318 | Clinical audit of all emergency surgical procedures whether undertaken in an operating theatre or another area (e.g. emergency resus room) is regularly undertaken | All | N/A | - |
| 319 | Named supervisory consultants are available to all non-consultant anaesthetists. Those they are supervising know their identity, location, and how to contact them. In situations where a trainee is remotely supervised, the trainee must contact their supervising consultant immediately who should attend as soon as is possible | All | N/A | - |
| 320 | The perioperative anaesthetic care of all patients is, at all times, led by a consultant anaesthetist. Clinical care may be delegated to a supervised, clinically competent trainee of sufficient seniority | All | N/A | - |
| 321 | Patient transfer is carried out to standards described by the AAGBI | All | N/A | - |
| 322 | A consultant in intensive care medicine reviews all emergency surgical admissions to the ICU within 12 h | All | N/A | - |
| 323 | A geriatrician assesses hip fracture patients within 72 h of admission | All | N/A | - |
| 324 | Anaesthetists offering perioperative analgesia services should provide, in collaboration with others as appropriate, patient and family education regarding their important roles in achieving comfort, reporting pain, and in proper use of recommended analgesic methods | All | 3b | - |

***Note.*** AAGBI = Association of Anaesthetists of Great Britain and Ireland; CSII = continuous subcutaneous insulin infusion; CFS = Clinical Frailty Scale; EPC = enhanced perioperative care; ERAS = enhanced recovery after surgery; OSA = obstructive sleep apnoea; PACU = post anaesthesia care unit; PONV = postoperative nausea and vomiting; SBYB = stop before you block; VTE = venous thromboembolism. **^NEW^** = indicators reported for use since January 2016.

**Table S3.** Indicators not clearly defined as process or structure with level of evidence

| **No.** | **Indicators not clearly defined as process or structure** | **Perioperative Phase** | **Level of evidence (studies using indicators as measures)** | **Level of evidence (studies evaluating validity of indicators)** |
| --- | --- | --- | --- | --- |
| 1 | Have preoperative assessment personnel had formal training in offering advice and guidance on exercise interventions? **^NEW^** | Pre-op | 5 | - |
| 2 | Diabetes specialist team was consulted prior to surgery? **^NEW^** | Pre-op | 2c | - |
| 3 | The decision for any therapeutic intervention, excluding diagnostic procedure, has been taken by a multidisciplinary team, including at least a gynaecologic oncologist or specialized gynaecologic surgeon dedicated to the management of gynaecological cancer, a radiologist, a medical or clinical oncologist, a pathologist, and a radiation oncologist specialized in the treatment of gynaecological cancers and with expertise in brachytherapy **^NEW^** | Pre-op | - | 5 |
| 4 | Treatment planned and reviewed at a multidisciplinary team meeting **^NEW^** | Pre-op | - | 2c |
| 5 | Percentage of patients, regardless of age, who receive anaesthesia services for same-day surgery with documented patient escort prior to the start of anaesthesia **^NEW^** | Pre-op | 5 | - |
| 6 | Availability of written policies for preoperative preparation (including all the following: fasting, investigations, blood typing, thromboprophylaxis, peri-operative diabetes management, and allergies) **^NEW^** | Pre-op | - | 1a |
| 7 | Percentage of anaesthetic assistants who have received training in SBYB process and access to continuing training opportunities **^NEW^** | Intra-op | 5 | - |
| 8 | Are staff trained on the appropriate use of TIVA and related equipment and is enough equipment available for use? **^NEW^** | Intra-op | 2c | - |
| 9 | Number of formal TIVA/TCI sessions attended per training level **^NEW^** | Intra-op | 2c | - |
| 10 | The recovery room staff are appropriately trained in all relevant aspects of postoperative care and are present in appropriate numbers | Intra-op | N/A | - |
| 11 | Proportion of anaesthetists aware of and able to identify local massive transfusion protocol **^NEW^** | Intra-op | 2a | - |
| 12 | Grade of most senior anaesthetic and surgical staff seeing patient pre-operatively & in theatre **^NEW^** | Intra-op | 2c | - |
| 13 | People having surgery for inflammatory bowel disease have it undertaken by a colorectal surgeon who is a core member of the inflammatory bowel disease multidisciplinary team | Intra-op | N/A | - |
| 14 | All records for anaesthesia and sedation contain the relevant part of the recommended anaesthetic dataset and are kept as a permanent document in the patient's record | Intra-op | N/A | - |
| 15 | Percentage of anaesthetists aware of the location and content of anaphylaxis treatment packs **^NEW^** | Intra-op | 2c | - |
| 16 | Percentage of anaesthetists aware of where the nearest glucagon and vasopressin are to be found **^NEW^** | Intra-op | 2c | - |
| 17 | Percentage of anaesthetists who know where to refer suspected anaphylaxis patients for further investigation **^NEW^** | Intra-op | 2c | - |
| 18 | Percentage of anaesthetists aware of time points to check serum tryptase **^NEW^** | Intra-op | 2c | - |
| 19 | Standards are addressed at induction **^NEW^** | Intra-op | 2c | - |
| 20 | Percentage of cases that have continuous monitoring between theatre and recovery feedback compliance to staff using run charts **^NEW^** | Intra-op | 2c | - |
| 21 | Percentage of core trainees who have logged the requisite number of TIVA/TCI cases **^NEW^** | Intra-op | 2c | - |
| 22 | The percentage of anaesthetists aware of what constitutes a comprehensive referral **^NEW^** | Intra-op | 2c | - |
| 23 | A blood-management guideline or protocol to reduce perioperative blood administered in case of hip replacement is present (yes/no): (as one component of ERAS process measures) **^NEW^** | Intra-op | - | 5 |
| 24 | Presence of a trained recovery room nurse **^NEW^** | Post-op | 5 | - |
| 25 | Percentage of patients admitted to the PACU out of hours where there are two members of staff present in the PACU until the patient is discharged **^NEW^** | Post-op | 2c | - |
| 26 | Regular education and training of PACU staff to national standards | Post-op | 5 | - |
| 27 | Percentage of patients whose recovery record contains the Association’s minimum dataset **^NEW^** | Post-op | 2c | - |
| 28 | Percentage of patients receiving critical care in recovery for more than four hours whose recovery record contains the Critical Care Minimum Dataset **^NEW^** | Post-op | 2c | - |
| 29 | Written postoperative pain management protocols **^NEW^** | Post-op | 4 | - |
| 30 | Trainings in postoperative pain management **^NEW^** | Post-op | 4 | - |
| 31 | Number/proportion of staff working in perioperative care settings who have completed training up to (and including) eLFH level 3 training or equivalent **^NEW^** | All | 5 | - |
| 32 | Number/proportion of staff working with patients living with frailty who have completed tier 1, 2, or 3 training **^NEW^** | All | 5 | - |
| 33 | Presence of a trained assistant **^NEW^** | All | 5 | - |
| 34 | Multidisciplinary team involved in all hip fracture patients > 70 years **^NEW^** | All | - | 2c |
| 35 | The patient was seen by all the appropriate staff? **^NEW^** | All | 2c | - |
| 36 | Surgical volume **^NEW^** | All | 2a | - |
| 37 | Case volume **^NEW^** | All | 2a | - |
| 38 | Volume of EVT procedures for stroke **^NEW^** | All | - | 2a |
| 39 | Procedure rate **^NEW^** | All | - | 2a |
| 40 | High hospital volume **^NEW^** | All | 2a | - |
| 41 | Procedures done in an operating theater, per 100,000 per year **^NEW^** | All | 2a | - |
| 42 | Hospital annual case volume | All | 1a | - |
| 43 | Surgical monthly/annual case volume by surgical speciality | All | 1a | - |

***Note.*** EVT = Endovascular thrombectomy; ERAS = enhanced recovery after surgery; PACU = post-anaesthesia care unit; SBYB = stop before you block; TIVA = total intravenous anaesthesia. **^NEW^** = indicators reported for use since January 2016.
